# Understanding Cation–Anion Ionic Bonding in Tetramethylammonium Salts: Insights from Density Functional Theory and X-ray Crystallography

**DOI:** 10.1021/acsomega.4c07265

**Published:** 2024-10-28

**Authors:** W. Christopher Hollinsed, Abigail Taber

**Affiliations:** Department of Chemistry and Biochemistry, James Madison University, Harrisonburg, Virginia 22807, United States

## Abstract

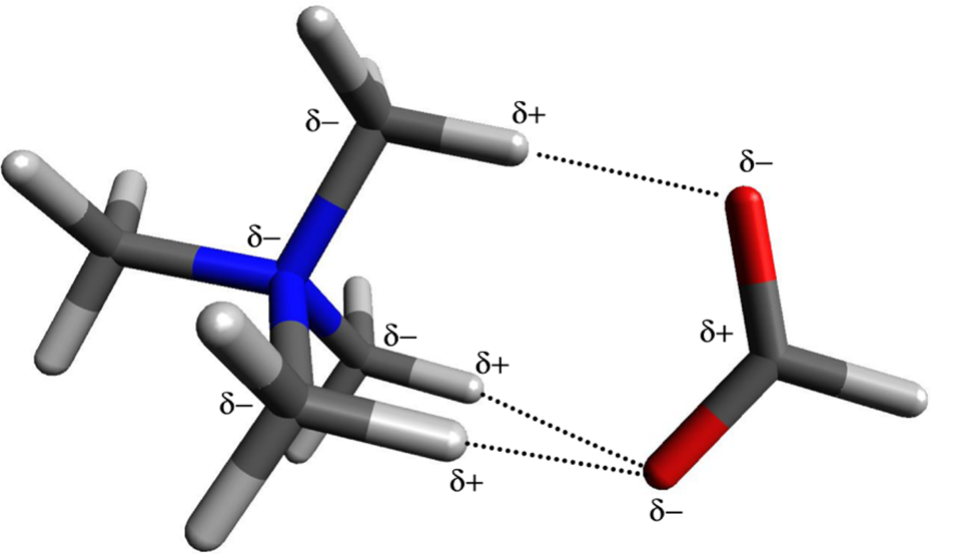

In describing the charge on tetraalkylammonium ions, a charge of +1 is usually assigned. This is both the actual charge as well as the “formal charge” which is usually written on the nitrogen atom to indicate the electron deficiency experienced by the nitrogen atom relative to the valence electrons. Nitrogen is the most electronegative atom in the ion. The results in this study show that despite the convention of writing the positive charge on nitrogen, the ion is polarized through the σ bond framework to present four tetrahedral faces, each having close to a full charge of +1 to any anionic species which approach the cation.

## Introduction

In the typical academic general chemistry course, students are taught to understand ammonium compounds through the application of their “formal charge”.^1,2^ For ammonium compounds this is a charge of positive 1. While the overall ion has a charge of +1, (which is written on nitrogen to reflect the implied electron deficiency relative to the valence electrons) it is not so clear how negative counterions are paired with the tetraalkylammonium ion given that the alleged positive charge on nitrogen is surrounded by hydrogen atoms. Most importantly, the convention that encourages us to write the positive charge on nitrogen is contradicted by the fact that nitrogen is the most electronegative element in the ion.

## Results and Discussion

### Tetramethylammonium Ion

#### Polarization through the Sigma Bonded Framework Leads to the Positive Charge on the Exterior of the Ion

**Figure 1:**
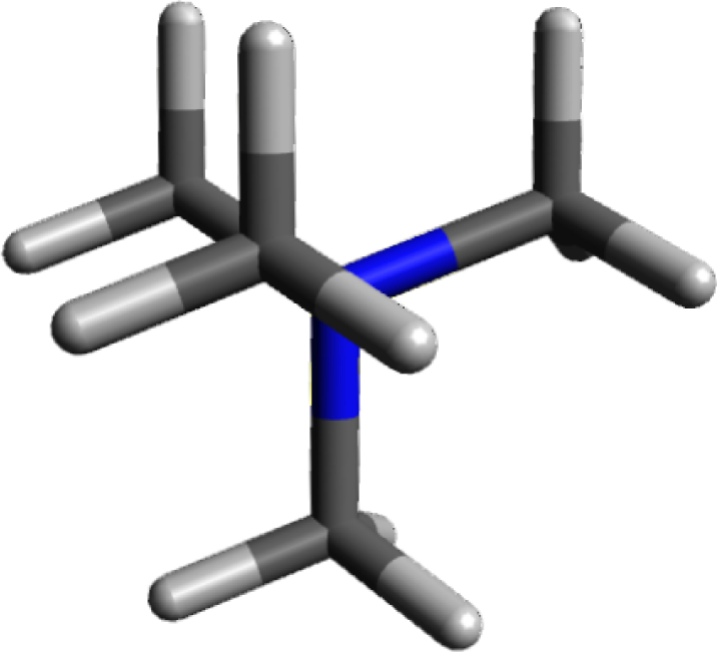
Calculated structure of tetramethylammonium ion.

Tetramethylammonium ion was calculated providing the optimized geometry and the NPA charges. The geometry is shown in Figure 1. As suspected, the central nitrogen atom possesses a significant amount of the electron density, with a charge of −0.344. However, the four carbon atoms have an even more negative charge of −0.475 each. The Pauling electronegativity^3^ of nitrogen is 3.04 and carbon is 2.55. Hydrogen, of which there are 12 in this ion has an electronegativity of 2.2 shows considerable electron deficiency. Each hydrogen atom has a charge of +0.270. The atoms are arranged as expected, in such a manner that decreases steric interactions.

**Figure 2:**
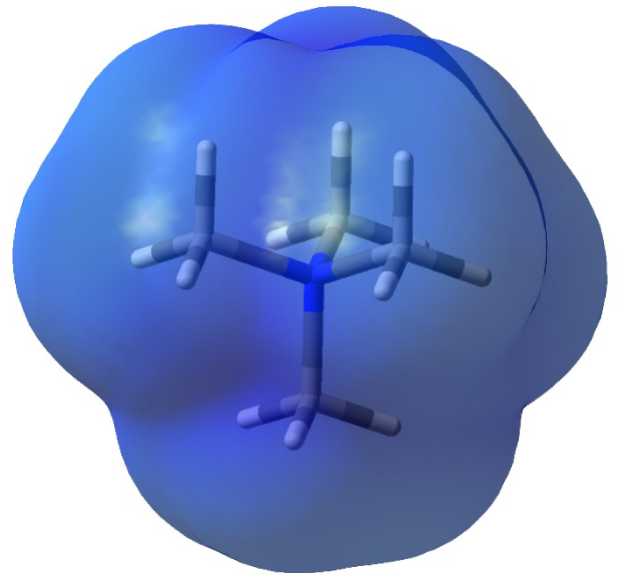
Electrostatic potential surface map of tetramethylammonium cation.

The carbons are arranged in a tetrahedral arrangement as expected for the sp^3^ hybridized nitrogen to which they are bonded. The hydrogens are similarly arranged in a tetrahedral arrangement resulting in an overall tetrahedron arrangement for the ion with each face consisting of 3 hydrogen atoms. An anion approaching such an ion will encounter a face having (3 × +0.270) = +0.810 as the charge. Moreover, each face has a comparable charge which means that a second negatively charged ion will encounter a comparable charge on a different face. Not only is there no reason to be concerned that there is no physical access to the formal charge “+1” of the interior nitrogen atom but there is plenty of positive charge on the outside of the ion available to interact with any anionic species available to form a charge balanced ion-pair. The intensity and arrangement of the positive and negative charges in the ion strongly suggests an important conclusion: *Polarization through the sigma bonded framework leads to the positive charge on the exterior of the ion.* In addition, the symmetry of this polarization indicates that the ion will present an electronically positive face regardless of how an anion approaches the cation. An electrostatic potential diagram of the tetramethylammonium cation is shown in Figure 2. Since blue represents electropositive potential, it is clear that the entire exterior of the cation is positively charged.

### Tetramethylammonium Chloride

**Figure 3:**
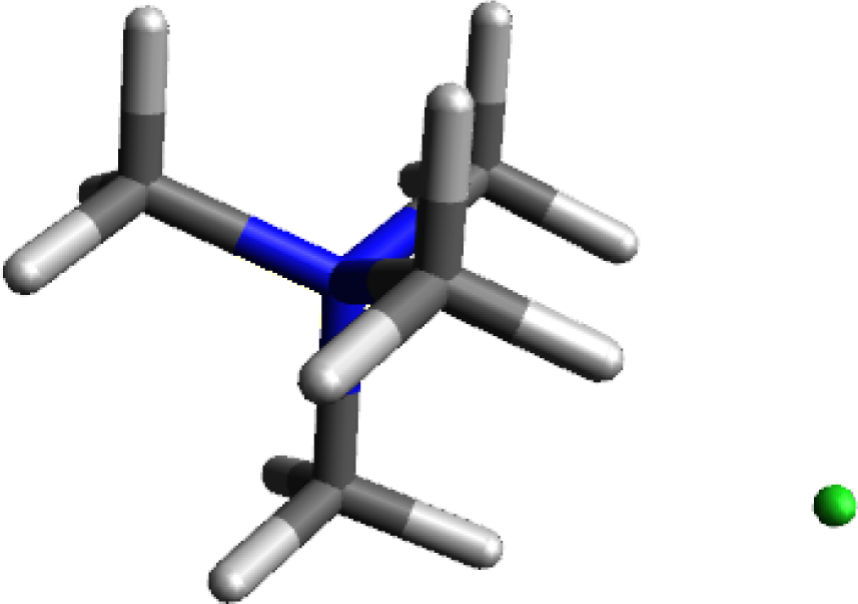
Calculated structure of tetramethylammonium chloride.

Once it was understood that the tetramethylammonium ion was polarized to be positive on the exterior, it made sense to calculate the ion pair with a typical counterion such as chloride. The structure of tetramethylammonium chloride is shown in Figure 3. The chloride ion itself was expected to have a full −1 charge but shows in this calculation a small amount of electron deficiency^4^ resulting in a charge of −0.889. The corresponding whole ion charge on the tetramethylammonium ion is +0.893 balancing the two opposite charges with a discrepancy of ∼+0.004 e. The charges on the hydrogens facing the chloride ion are even more electropositive than in the parent ion. Each hydrogen atom on the chloride face has a charge of +0.289 for a total face charge of +0.867. The distance between the chloride and each of the hydrogens is 2.39 Å.

### Tetramethylammonium Chloride X-ray Crystal Structure

A crystal structure^5^ of tetramethylammonium chloride shows a somewhat different orientation in the unit cell. In this structure chlorine seems to be paired with a single methyl group. (Since all of the hydrogens in a single methyl group are identical to the 3 hydrogens in the face orientation the charge balance is the same.) Closer examination of the crystal structure shows methyl orientation on two sides of the chloride and face orientation on two other sides with two tetramethylammonium ions. Each chlorine is surrounded by 4 tetramethylammonium ions, two with methyl orientation, two with face orientation. The distance between the methyl orientation hydrogens and the chlorine averages to 3.41 Å. The closer contact, however, is between the chlorine and face protons (3.13 Å). These distances are significantly longer than in the calculation, however there are four molecules for every chloride ion available to balance charge.

### Tetramethylammonium Hydroxide

**Figure 4:**
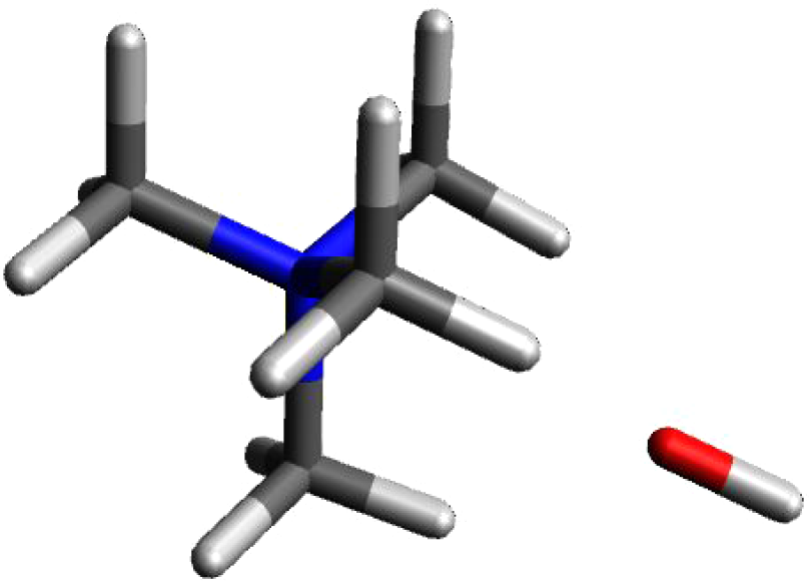
Calculated structure of tetramethylammonium hydroxide.

Figure 4 shows the calculated structure for tetramethylammonium hydroxide. Hydroxide ion, similar to chloride should have a charge of −1. The electronegative oxygen is able to polarize the O–H bond in such a manner that the calculated charge for the hydroxide oxygen is −1.322, The hydrogen of the −OH group has a charge of +0.432. The total charge for the hydroxide ion comes to −0.884, similar to the deficit observed in the calculation for chloride.

### Tetramethylammonium Formate

**Figure 5:**
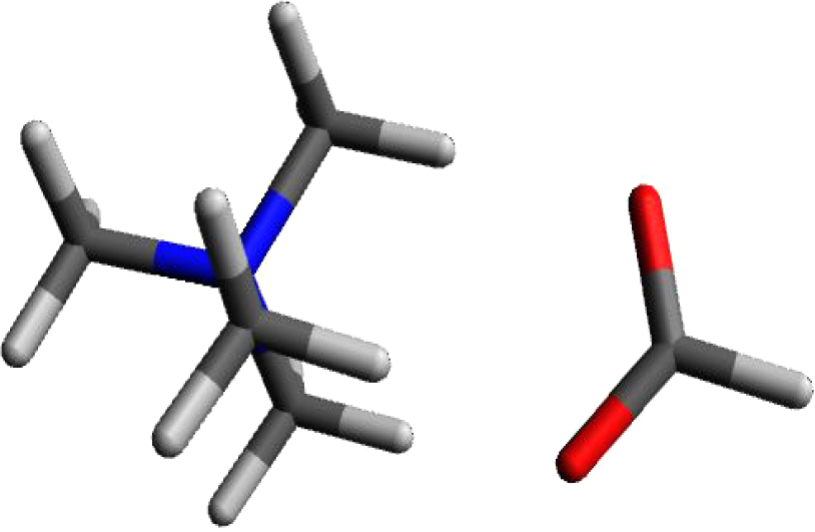
Calculated structure of tetramethylammonium formate.

Figure 5 shows the calculated structure for tetramethylammonium formate. In the case of tetramethylammonium formate, the anion has two oxygens sharing a single negative charge. The calculated structure has one oxygen in a head-to-head arrangement with one hydrogen atom on the tetramethylammonium ion and the other oxygen bifurcating the two hydrogens which comprise the same cationic face of the tetramethylammonium cation. Remarkably, the polarization of the two carboxylate ion carbon–oxygen bonds leads to a combined charge for the two oxygens which is more than the −1 charge normally assigned to the anion. The additional electron density is pulled out of the carboxyl carbon and its attached proton. The combined oxygen charge is −1.587, the carboxyl carbon has a positive charge of +0.639. The overall charge for the formate ion (O=CH–O^–^) is −0.897, similar to the charge of −0.867 for chloride ion discussed above and the value of −0.884 for hydroxide ion.

### Tetramethylammonium Nitrite

**Figure 6:**
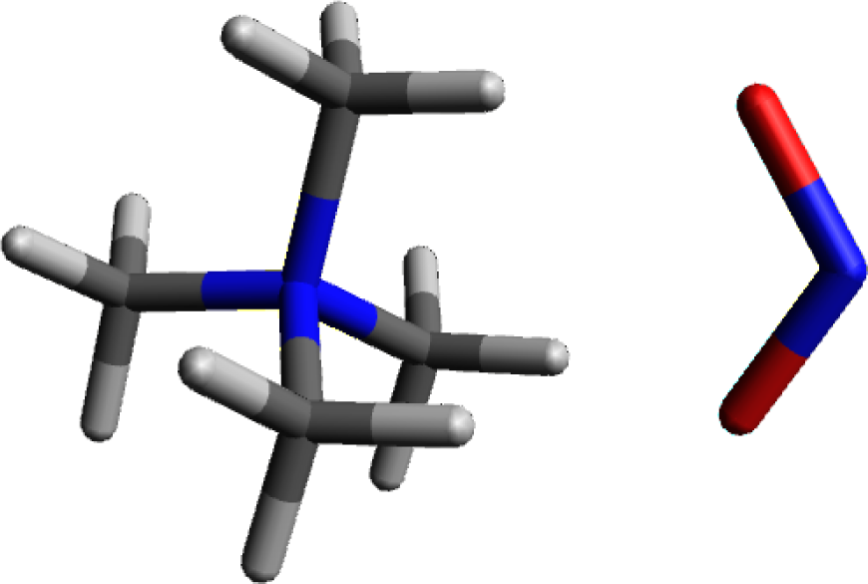
Calculated structure of tetramethylammonium nitrite.

Figure 6 shows the calculated structure for tetramethylammonium nitrite. Nitrite ion (NO_2_^–^) has a similar geometric structure to formate ion. Once again, there is a hydrogen–oxygen pairing which is head-to-head and a second set of pairings where the other oxygen is equidistant between the other two hydrogens on the cationic face. Similar to formate, the oxygens are bonded to a less electronegative element, in this case nitrogen. The combined charge for the two oxygens is more than the −1 charge which would be expected. The additional electron density is pulled out of the nitrite nitrogen atom. The combined oxygen charge is −1.16, the nitrite nitrogen atom has a positive charge of +0.277. The overall charge on nitrite ion is −0.882, comparable to chloride, hydroxide and formate.

### Tetramethylammonium Nitrate

**Figure 7:**
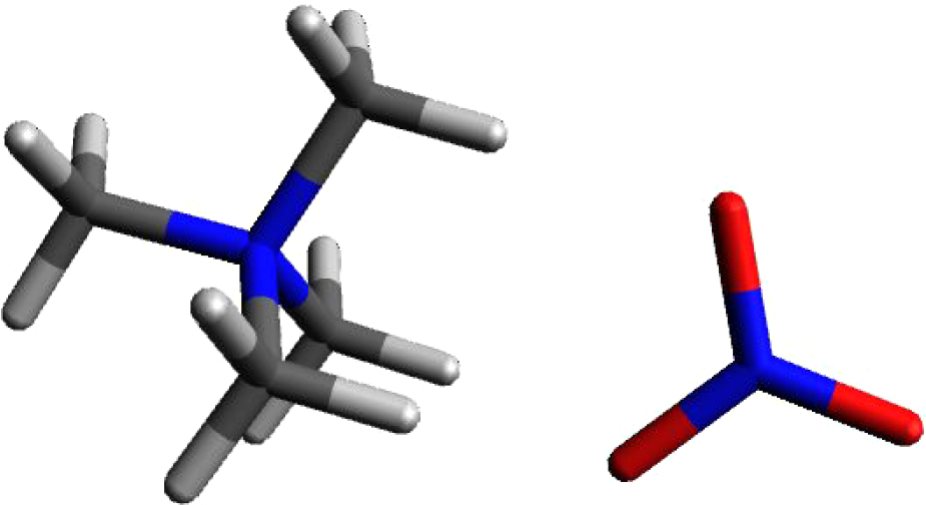
Calculated structure of tetramethylammonium nitrate – edge–face orientation.

It was initially presumed that nitrate would ionically bond in the same edge–face form that formate and nitrite ion-paired. The initial geometry for the calculation used an edge to face structure. The edge–face calculation for tetramethylammonium nitrate is shown in Figure 7. As expected, the result was one oxygen in a head-to-head relationship with a cationic face hydrogen and a second oxygen ionically paired equally to the remaining two hydrogens on the same face. The polarization in the nitrate ion is similar to that in formate and nitrite which suggests that this is a general phenomenon. The combined charge on the two oxygens involved in ion pairing with the cation is −1.176. The apical oxygen, which is not involved in ion pairing in this salt, also draws electron density away from the central nitrogen. The overall charge on nitrate is −0.920. The charge on the central nitrogen in the nitrate ion is +0.698. Charges for TMA^+^NO_3_^–^ are +0.918 for the tetramethylammonium ion and −0.922 for the nitrate anion.

### Nitrate Orientation in Crystal Structure

An X-ray crystal structure of tetramethylammonium nitrate^6^ shows a completely different orientation for the nitrate ion in this ion-pair. In the unit cell for this structure, the nitrate ion is in a face orientation with each of the three oxygens on the nitrate ion midway between each of the three hydrogens on the tetramethylammonium face. However, it is important to note that in the solid, crystalline phase, there are many additional possibilities for charge stabilizing close contacts.

Upon closer examination of the structure, it became clear that each oxygen is supported by other tetramethylammonium ions. In fact, each nitrate ion is surrounded by roughly 6 tetramethylammonium ions with contacts shorter than 3 Å to each one. The face orientation is only one orientation that nitrate can adopt. In this structure, the apical methyl group (the one not involved in face bonding) serves in the role of methyl bonding as was observed in the tetramethylammonium chloride ion-pair discussed previously.

We carried out a face–face calculation to compare to the edge–face calculation. However, it is clear that in the crystalline state there are many energetically favorable options for charge neutralization that will not be seen in a single ion-pair calculation.

### Tetramethylammonium Nitrate

#### Face–Face Calculation

**Figure 8:**
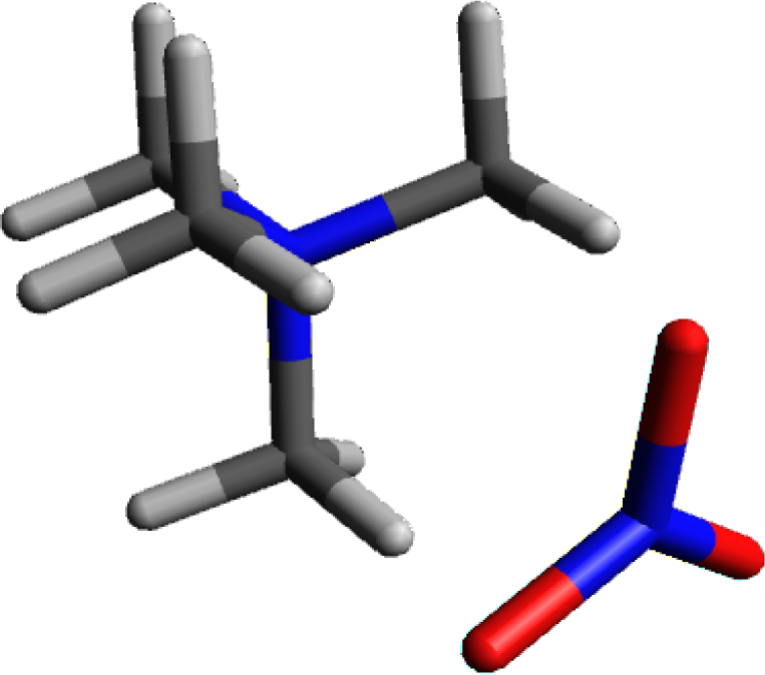
Calculated structure of tetramethylammonium nitrate – face–face orientation.

A calculation in the face–face configuration (Figure 8) for TMA^+^NO_3_^–^ shows structural features comparable to the crystal structure. The ideal structure would have a dihedral angle O–N–N–C between the NO bond on nitrate and the NC bond in the cation of 60°. Both the calculation and the crystal structure give dihedral angles, close to 60°. The dihedral angle in the crystal structure is 61.2° and 61.8° for the calculation. The distances between the oxygens on the nitrate ion and hydrogens on the ammonium cation are on average ∼0.5 Å longer in the crystal structure (average of 2.34 Å, for the calculation, and 2.86 Å for the crystal structure) than in the calculation. However, as noted, each of the oxygens in the crystal structure is ionically paired with electropositive hydrogens from other tetramethylammonium cations.

As in the edge nitrate, nitrite and formate ions, the combined charges on the oxygen atoms are larger than the assigned charge of −1 and in the case of the face nitrate they add up to −1.661. Once again, the additional electron density comes from the central nitrogen atom which has a charge of +0.667. In the face–face configuration, each of the three oxygens is involved in ion pairing.

#### Calculated Energies

Based on the calculated energies, for the edge–face and face–face conformations for TMA^+^NO_3_^–^ the edge–face conformation is more stable by 10.4 kJ (2.5 kcal/mol). This is a relatively small difference and underscores the fact that unit cell data from the crystal structure where each ion is stabilized by many adjacent counterions does not significantly change the conclusions based on the edge calculation.

### Tetramethylammonium Carbonate

#### Edge–Face Conformation

**Figure 9:**
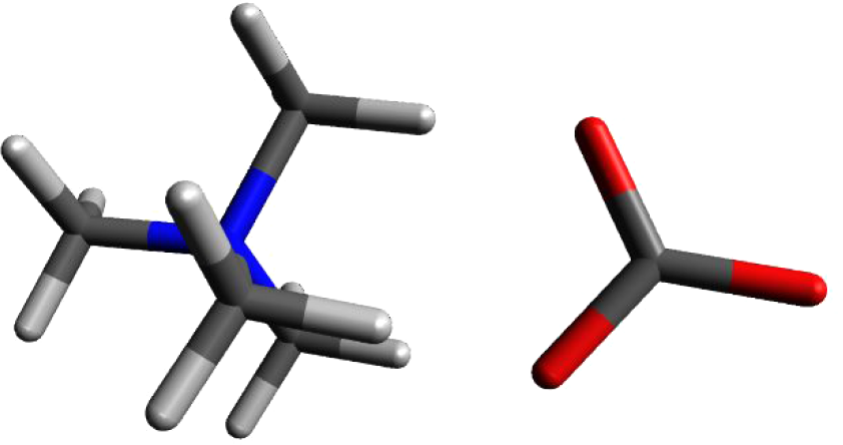
Calculated structure of tetramethylammonium carbonate, edge–face orientation.

**Figure 10:**
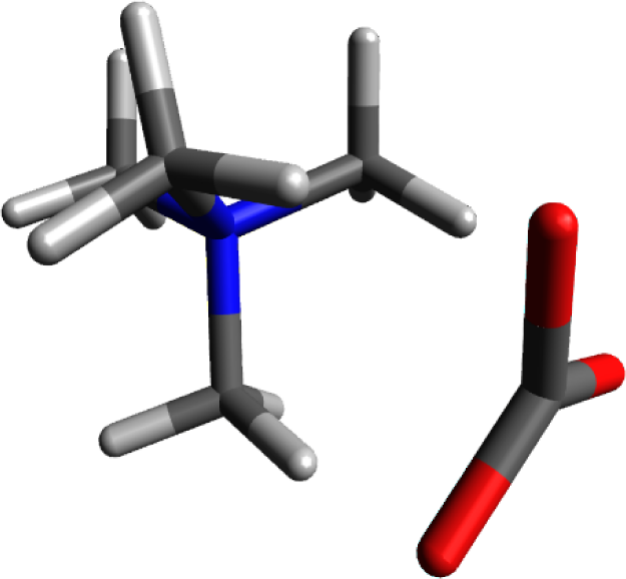
Calculated structure of tetramethylammonium carbonate, face–face orientation.

The edge–face orientation (Figure 9) and the face–face orientation (Figure 10) were calculated for the ion pair between tetramethylammonium ion and carbonate ion. Since carbonate has an assigned charge of −2, the ion pair has an overall charge of −1.

Carbonate ion is trigonal planar. As in the edge–face conformation for TMA^+^NO_3_^–^, the structure has a single oxygen head-to-head with a hydrogen atom on the tetramethylammonium ion face and a second oxygen midway between the remaining two hydrogens on the same face.

### Tetramethylammonium Carbonate

#### Face–Face Conformation

In both the X-ray crystal structure and the calculation for TMA–NO_3_ the oxygens are very close to the center of the line between each pair of the face hydrogens on the cation. The dihedral angles are 58.8° for the crystal structure and 58.2° for the calculated structure, close to the ideal angle of 60°. The calculation for carbonate shows a somewhat different arrangement. Carbonate has an orientation which is between the head-to-head orientation and the bisected orientation. The dihedral angle is 35.3°. In addition, the methyl groups are twisted slightly so that the hydrogens can be closer to the oxygen. The closest contacts for the O–H distances are 1.79 Å, comparable to the bisected O–H distance in the edge–face conformation.

#### Calculated Energies

Calculated energies for the edge–face and face–face conformations for TMA^+^CO_3_^–^ show that the edge–face conformation is more stable by 8.4 kJ (2.0 kcal/mol). Once again this is a relatively small difference easily overcome by solvent effects in the solution phase and packing forces in the solid state.

### Ion-Pairing with Neutral Molecules

#### Tetramethylammonium Ion and Nitromethane

**Figure 11:**
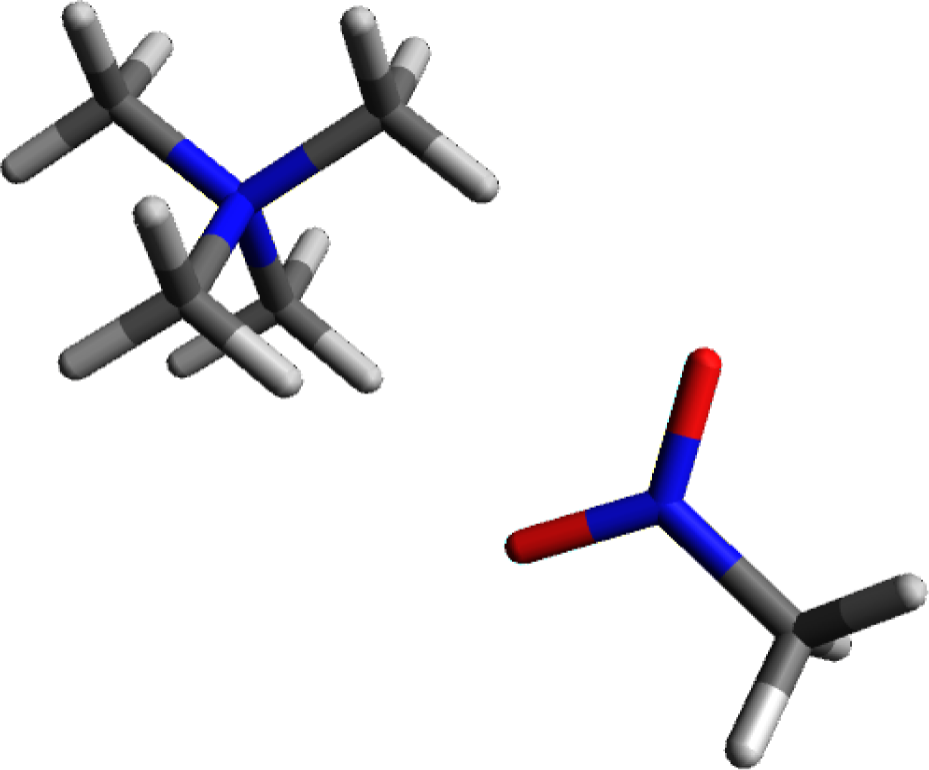
Calculated structure of tetramethylammonium ion complexed to nitromethane, edge–face orientation.

The nitro group attached to a methyl group has a similar configuration as the nitrite ion. The nitro group is attracted to the tetramethylammonium ion in a similar fashion (see Figure 11). One of the nitro group oxygens is in a head-to-head arrangement with one of the hydrogens, the other oxygen bisects the space between the remaining cation face hydrogens. Similar to formate and nitrite, the oxygens are bonded to the less electronegative nitrogen. The combined charge for the two oxygens is −0.774.

The additional electron density is pulled out of the nitrate nitrogen atom which has a charge of +0.498. The overall charge on nitrate group is −0.276. As expected, nitromethane is polarized even though as a molecule it is electrically neutral. The distance between the ammonium nitrogen and the methyl nitrogen is 4.47 Å, somewhat longer than the comparable distance between the nitrogens in the ionically bonded tetramethylammonium nitrite of 4.13 Å. A calculation of the complex between tetraethylammonium ion and nitrobenzene has been carried out by Fry.^7^ The same forces that cause tetraalkylammonium salts to form are also available to form complexes with neutral molecules of comparable geometry. Although a clearly weaker “ion-pair”, the tetramethylammonium ion should clearly be able to pair with a compound like nitromethane which has a polar nitro group similar to the nitrite and nitrate ions calculated above. It is expected that the forces holding the ion–neutral pair together will be significantly weaker than the ion–ion pair complexes calculated above.

## Conclusions

Polarization through the sigma bonded framework in tetraalkylammonium ions leads to the positive charge on the exterior of the ion.

The more electronegative elements carbon and nitrogen are responsible for the polarization.

The overall charge on these ions is assigned as +1 and in general, each face of the tetrahedrally shaped ion presents about +1 charge to any anionic species encountered, through the attraction to three (3) polarized hydrogen atoms.

There is “leakage” of charge from the anionic ions to the cationic ion resulting in slightly lower charge on the anionic side and correspondingly slightly lower positive charge on the cationic side. In all cases the positive and negative charges matched such that there is no residual charge on the ion pair. Since ionic bonding requires orbital–orbital interaction in order to form the attachment, it is not surprising that such charge leakage occurs.

On the anionic ions, polarization takes place as well, specifically between oxygen and atoms with lesser electronegativity. For example, each oxygen in formate ion, which would be assigned to have −0.5e for charges actually has −0.814 and −0.773 as charges. The oxygens draw electron density away from the carboxyl carbon which then has a charge of +0.639. This suggests the oxygens which are actually involved in the formation of the ionic bond may form a stronger bond with the hydrogen atoms on the tetramethylammonium cation than would have otherwise been expected.

In this work we limited the calculations to tetramethylammonium ions. Longer chain lengths have been considered previously by Fry^7^ The results are comparable to the work done here.

### Computational Methods

In this work insights and enhanced understanding of ion-pairing in these systems was developed through computational chemistry methods, specifically Density Functional Theory (DFT)^8^ using the B3LYP^9^ functional with the 6-311G+(d,p) basis set^10^ by using the program Gaussian 03.^11^ Initial geometries were determined using the semiempirical program, PM3^12^ and refined using the above technique. Charges (electron populations) calculated are the Natural Population Analysis (NPA) charges based on the Natural Bond Orbital (NBO) method of Weinhold et al.^13^ Numbers and associated charge values as well as atomic coordinates and calculated energies are included in the Supporting Information.

## Data Availability

Gaussian input and output files are available from Zenodo: https://doi.org/10.5281/zenodo.12802980.

## Supporting Information Available

Geometric coordinates, numbering system, and natural population analysis charges as well as energies for each calculated structure (PDF)
