# Supplementary material for: Understanding Cation–Anion Ionic Bonding in Tetramethylammonium Salts: Insights from Density Functional Theory and X-ray Crystallography
Source: ACS Omega. 2024 Oct 28;9(45):45342–7. doi: 10.1021/acsomega.4c07265 (PMC11561762; doi:10.1021/acsomega.4c07265)
Supplement: Supplementary file 1 — ao4c07265_si_001.pdf [file ao4c07265_si_001.pdf]

Supporting Information

Understanding Cation-Anion Ionic Bonding in Tetramethylammonium Salts: Insights from Density Functional Theory and X-Ray Crystallography.

*W. Christopher Hollinsed** *and Abigail Taber*, Department of Chemistry and Biochemistry, James Madison University, Harrisonburg, VA 22807

***Corresponding author:** W. Christopher Hollinsed Email: hollinwc@jmu.edu

Below are compound numbering schemes for each compound, calculated atomic coordinates for each molecule and/or ion pair, Natural Population Analysis (NPA) charges for each molecule and/or ion pair, total energy calculated.

	Compound	Numbering	Charges	Atomic Coordinates	(NPA) Charges
1	Tetramethylammonium (TMA) cation	Figure S1	Figure S2	Table S1	Table S2
2	TMA Chloride	Figure S3	Figure S4	Table S3	Table S4
3	TMA Hydroxide	Figure S5	Figure S6	Table S5	Table S6
4	TMA Formate	Figure S7	Figure S8	Table S7	Table S8
5	TMA Nitrite	Figure S9	Figure S10	Table S9	Table S10
6	TMA Nitrate Edge	Figure S11	Figure S12	Table S11	Table S12
7	TMA Nitrate Face	Figure S13	Figure S14	Table S13	Table S14
8	TMA Carbonate Edge	Figure S15	Figure S16	Table S15	Table S16
9	TMA Carbonate Face	Figure S17	Figure S18	Table S17	Table S18
10	TMA Nitromethane	Figure S19	Figure S20	Table S19	Table S20

1. Tetramethylammonium cation

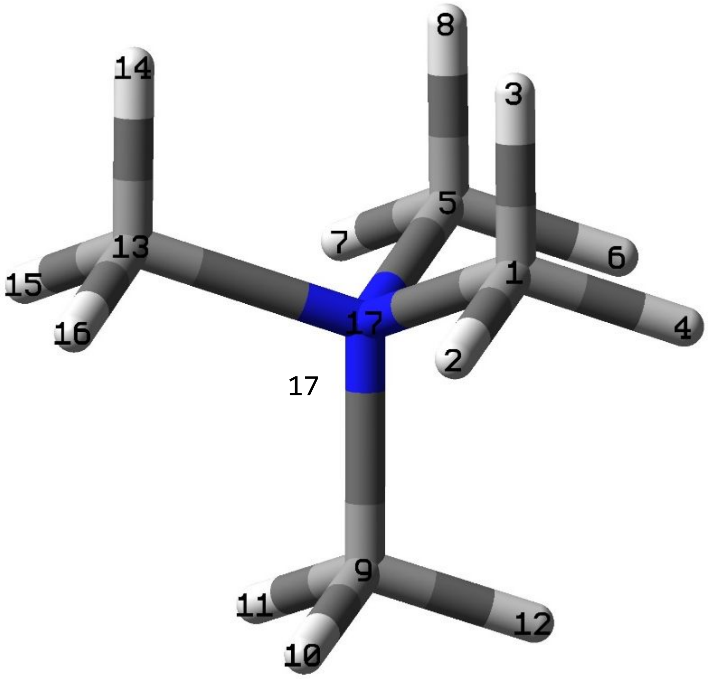

Figure S1. Numbering scheme for atoms in tetramethylammonium cation corresponding to the tables with geometric coordinates and NPA charges.

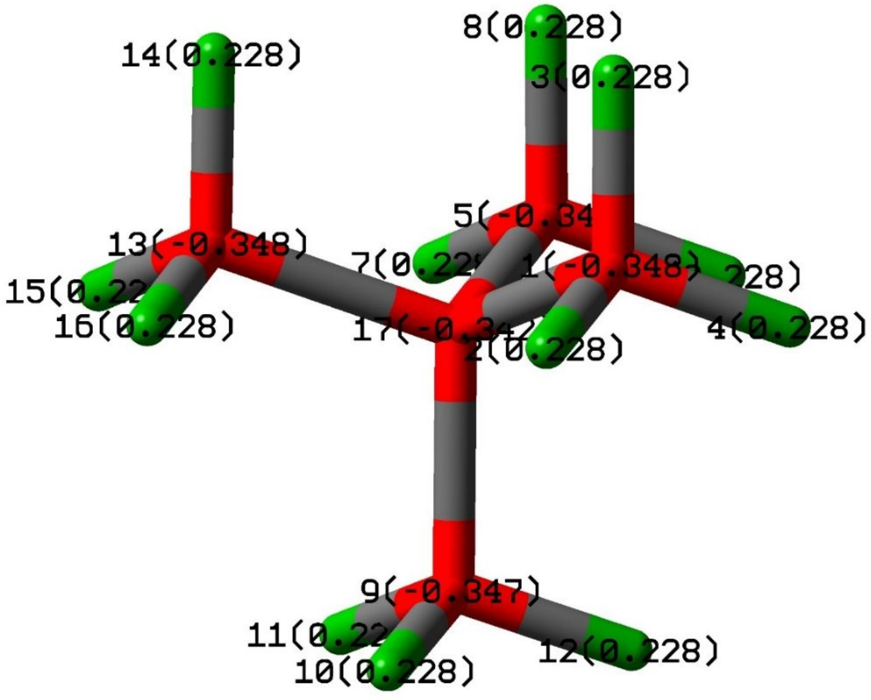

Figure S2. Numbering scheme for atoms in tetramethylammonium cation NPA charges in parentheses, RED =partial negative charge, GREEN = partial positive charge

Table S1: Atomic Coordinates:

Center Number	Atomic Number	Atomic Type	Coordinates (Angstroms) X	Y	Z
1	6	0	0.195291	1.131556	-0.977283
2	1	0	-0.606555	1.855658	-0.843017
3	1	0	0.170984	0.731824	-1.989667
4	1	0	1.158370	1.601283	-0.784773
5	6	0	1.107191	-1.009302	-0.171755
6	1	0	2.062641	-0.517808	0.004042
7	1	0	0.964491	-1.815137	0.546309
8	1	0	1.071320	-1.403193	-1.186089
9	6	0	0.026966	0.541130	1.407065
10	1	0	-0.776854	1.266437	1.521026
11	1	0	-0.112136	-0.282996	2.104657
12	1	0	0.988995	1.019769	1.582216
13	6	0	-1.329454	-0.663374	-0.257988
14	1	0	-1.337082	-1.055730	-1.273523
15	1	0	-1.463206	-1.474347	0.455974
16	1	0	-2.120935	0.074413	-0.137256
17	7	0	0.000000	-0.000034	-0.000018

Table S2: Natural Population Analysis Charges:

Atom	No	Natural Charge	Natural Population Core	Valence	Rydberg	Total
C	1	-0.34759	1.99930	4.33407	0.01422	6.34759
H	2	0.22767	0.00000	0.77130	0.00103	0.77233
H	3	0.22778	0.00000	0.77119	0.00103	0.77222
H	4	0.22762	0.00000	0.77135	0.00103	0.77238
C	5	-0.34752	1.99930	4.33400	0.01422	6.34752
H	6	0.22766	0.00000	0.77131	0.00103	0.77234
H	7	0.22769	0.00000	0.77128	0.00103	0.77231
H	8	0.22768	0.00000	0.77129	0.00103	0.77232
C	9	-0.34749	1.99930	4.33397	0.01422	6.34749
H	10	0.22766	0.00000	0.77131	0.00103	0.77234
H	11	0.22762	0.00000	0.77135	0.00103	0.77238
H	12	0.22771	0.00000	0.77126	0.00103	0.77229
C	13	-0.34752	1.99930	4.33400	0.01422	6.34752
H	14	0.22765	0.00000	0.77132	0.00103	0.77235
H	15	0.22771	0.00000	0.77126	0.00103	0.77229
H	16	0.22767	0.00000	0.77130	0.00103	0.77233
N	17	-0.34200	1.99951	5.33612	0.00638	7.34200
* Total *		1.00000	9.99672	31.92767	0.07561	42.00000

Energy Calculated: E(RB+HF-LYP) = -214.22319864 a.u.

2. Tetramethylammonium Chloride

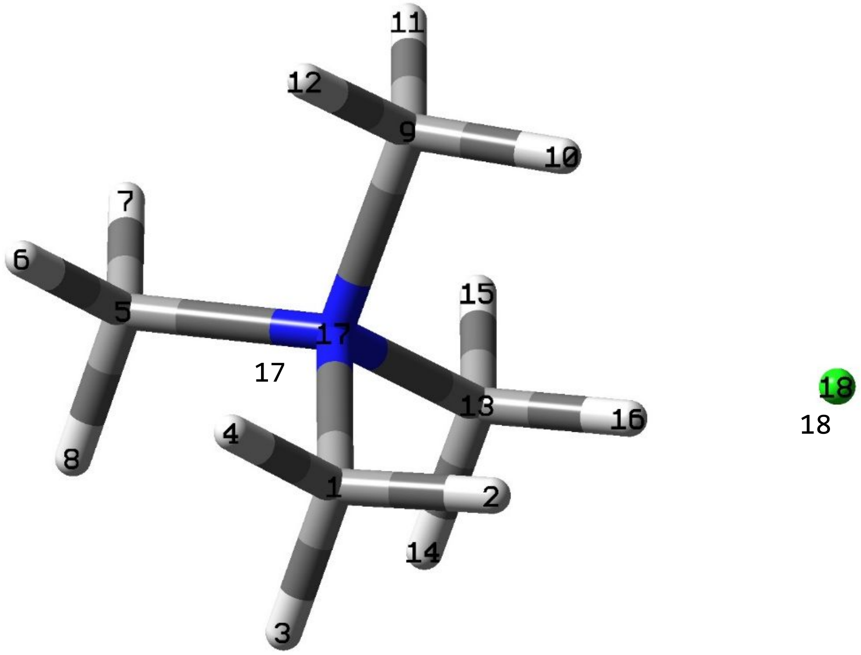

Figure S3. Numbering scheme for atoms in tetramethylammonium chloride corresponding to the tables with geometric coordinates and NPA charges.

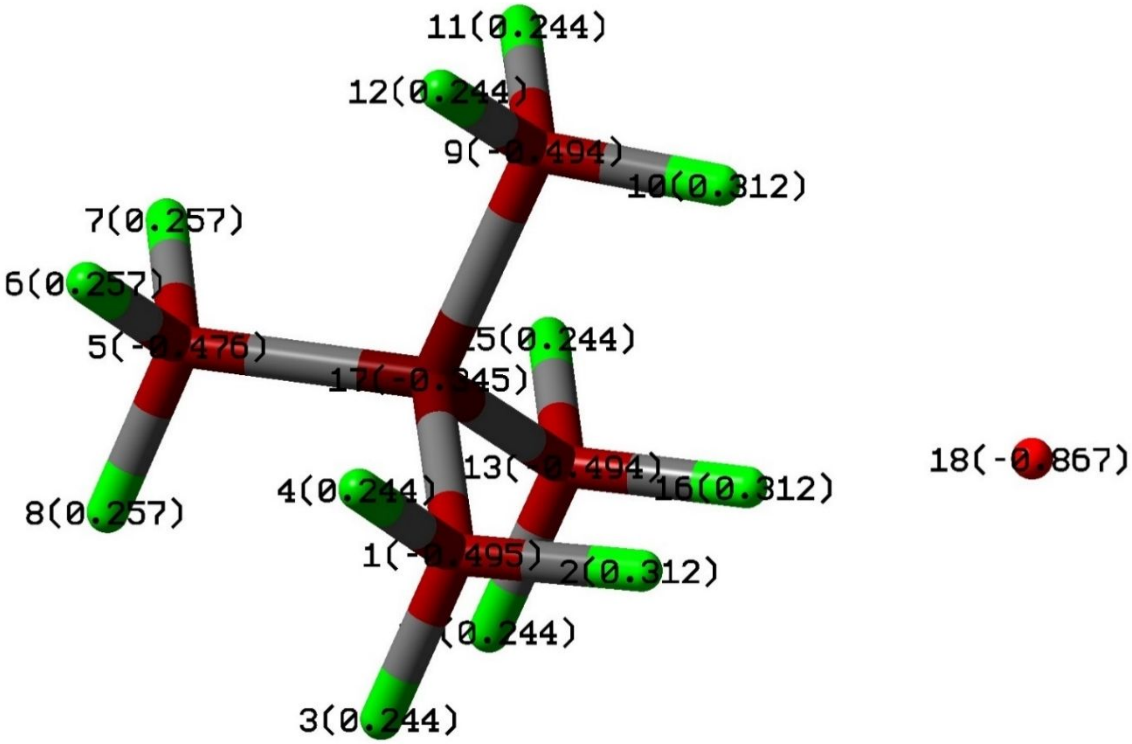

Figure S4. Numbering scheme for atoms in tetramethylammonium chloride, NPA charges in parentheses, RED =partial negative charge, GREEN = partial positive charge

Table S3: Tetramethylammonium Chloride Atomic Coordinates

Standard orientation:

Center Number	Atomic Number	Atomic Type	Coordinates (Angstroms) X	Y	Z
1	6	0	-0.502342	-0.299463	1.387402
2	1	0	0.594612	-0.282009	1.324889
3	1	0	-0.868780	-1.282551	1.687082
4	1	0	-0.876072	0.467295	2.067861
5	6	0	-2.521658	-0.000940	0.001709
6	1	0	-2.878050	0.763772	0.693272
7	1	0	-2.880338	0.214405	-1.005791
8	1	0	-2.877797	-0.981874	0.319486
9	6	0	-0.505707	1.352218	-0.434993
10	1	0	0.591247	1.293262	-0.420751
11	1	0	-0.881130	1.557340	-1.438849
12	1	0	-0.872560	2.102233	0.267427
13	6	0	-0.504455	-1.051899	-0.954204
14	1	0	-0.874963	-2.024615	-0.626971
15	1	0	-0.875330	-0.822034	-1.954326
16	1	0	0.592423	-1.005350	-0.912623
17	7	0	-1.025688	-0.000004	-0.000117
18	17	0	2.558325	0.000039	0.000037

Table S4. Tetramethylammonium Chloride NPA Charges

Summary of Natural Population Analysis:

Atom	No	Natural Charge	Natural Population Core	Valence	Rydberg	Total
C	1	-0.49461	1.99949	4.48045	0.01467	6.49461
H	2	0.31185	0.00000	0.68354	0.00462	0.68815
H	3	0.24432	0.00000	0.75472	0.00096	0.75568
H	4	0.24415	0.00000	0.75489	0.00096	0.75585
C	5	-0.47586	1.99953	4.46412	0.01221	6.47586
H	6	0.25685	0.00000	0.74210	0.00106	0.74315
H	7	0.25684	0.00000	0.74211	0.00106	0.74316
H	8	0.25688	0.00000	0.74206	0.00106	0.74312
C	9	-0.49446	1.99949	4.48033	0.01464	6.49446
H	10	0.31189	0.00000	0.68352	0.00458	0.68811
H	11	0.24420	0.00000	0.75484	0.00096	0.75580
H	12	0.24438	0.00000	0.75466	0.00096	0.75562
C	13	-0.49449	1.99949	4.48035	0.01465	6.49449
H	14	0.24426	0.00000	0.75479	0.00096	0.75574
H	15	0.24430	0.00000	0.75475	0.00096	0.75570

H	16	0.31185	0.00000	0.68355	0.00460	0.68815
N	17	-0.34489	1.99955	5.33055	0.01479	7.34489
Cl	18	-0.86745	9.99999	7.86543	0.00204	17.86745
==						
* Total *		0.00000	19.99753	39.90675	0.09572	60.00000

3. Tetramethylammonium hydroxide

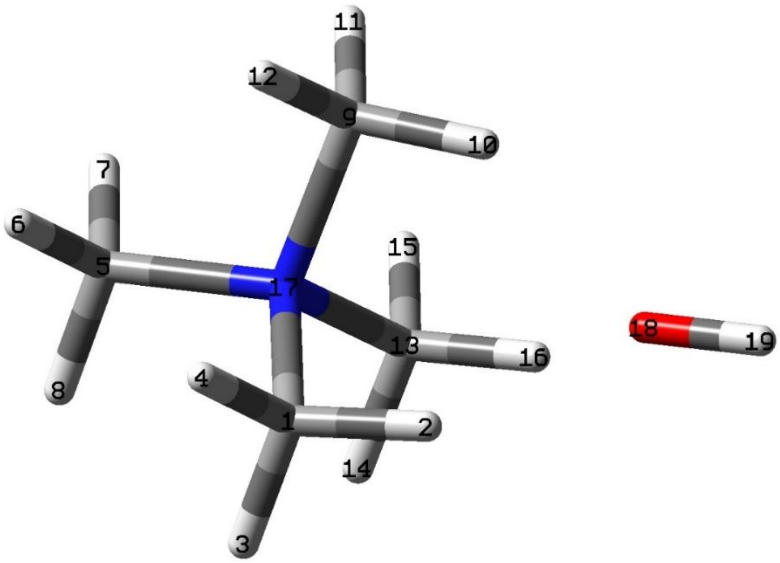

Figure S5. Numbering scheme for atoms in tetramethylammonium hydroxide corresponding to the tables with geometric coordinates and NPA charges.

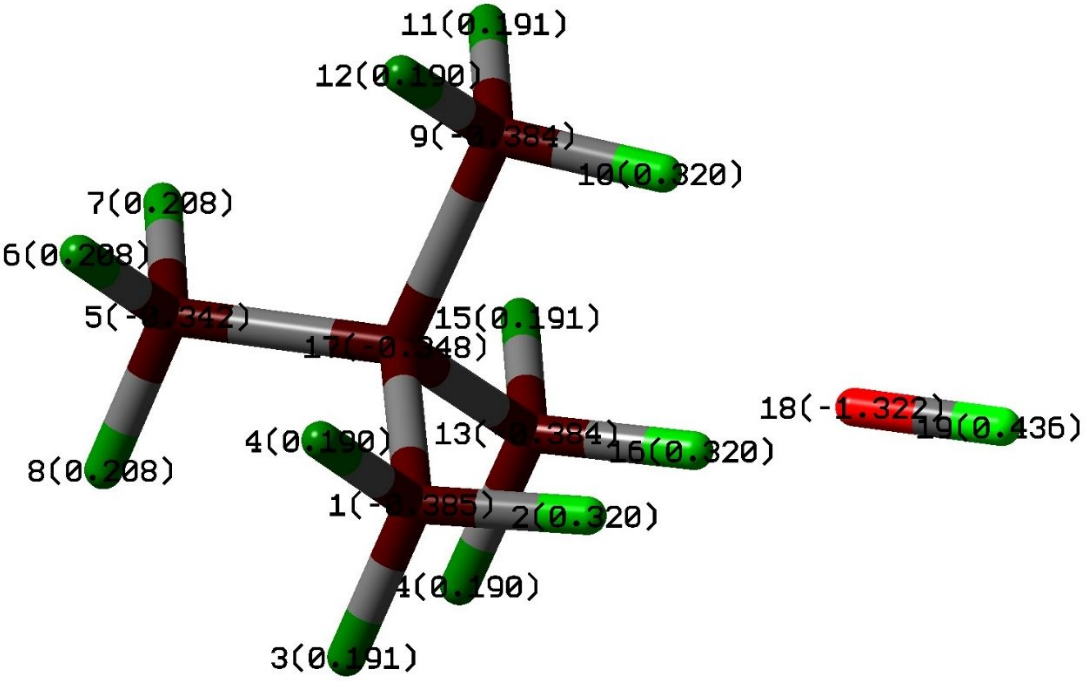

Figure S6. Numbering scheme for atoms in tetramethylammonium chloride, NPA charges in parentheses, RED =partial negative charge, GREEN = partial positive charge.

Table S5 Tetramethylammonium hydroxide Atomic Coordinates

Standard orientation:

Center Number	Atomic Number	Atomic Type	Coordinates (Angstroms) X	Y	Z
1	6	0	-0.022154	-0.313626	1.381543
2	1	0	1.081110	-0.287842	1.284153
3	1	0	-0.393140	-1.297862	1.669941
4	1	0	-0.398790	0.444896	2.068971
5	6	0	-2.042303	0.000681	-0.001089
6	1	0	-2.402021	0.755646	0.697910
7	1	0	-2.400385	0.228547	-1.005066
8	1	0	-2.401824	-0.982381	0.302814
9	6	0	-0.020307	1.353448	-0.418751
10	1	0	1.082583	1.259312	-0.384350
11	1	0	-0.388768	1.565491	-1.423061
12	1	0	-0.397864	2.097993	0.283286
13	6	0	-0.021266	-1.040519	-0.961155
14	1	0	-0.399795	-2.014195	-0.647541
15	1	0	-0.390194	-0.799328	-1.958615
16	1	0	1.081489	-0.970910	-0.889490
17	7	0	-0.552049	0.000340	-0.000050
18	8	0	2.429780	-0.000144	-0.000221
19	1	0	3.389886	-0.000488	-0.000117

Table S6 Tetramethylammonium hydroxide NPA Charges

Summary of Natural Population Analysis:

Atom	No	Natural Charge	Natural Population Core	Valence	Rydberg	Total
C	1	-0.38457	1.99929	4.36989	0.01539	6.38457
H	2	0.32031	0.00000	0.67779	0.00190	0.67969
H	3	0.19057	0.00000	0.80820	0.00123	0.80943
H	4	0.19044	0.00000	0.80832	0.00124	0.80956
C	5	-0.34156	1.99930	4.32918	0.01308	6.34156
H	6	0.20826	0.00000	0.79043	0.00131	0.79174
H	7	0.20830	0.00000	0.79039	0.00131	0.79170
H	8	0.20824	0.00000	0.79045	0.00131	0.79176
C	9	-0.38445	1.99929	4.36976	0.01540	6.38445
H	10	0.32035	0.00000	0.67775	0.00190	0.67965
H	11	0.19058	0.00000	0.80818	0.00123	0.80942
H	12	0.19039	0.00000	0.80838	0.00124	0.80961
C	13	-0.38419	1.99929	4.36951	0.01539	6.38419
H	14	0.19032	0.00000	0.80844	0.00124	0.80968
H	15	0.19059	0.00000	0.80818	0.00123	0.80941
H	16	0.32023	0.00000	0.67787	0.00190	0.67977
N	17	-0.34761	1.99951	5.33955	0.00855	7.34761
O	18	-1.32246	1.99984	7.31488	0.00773	9.32246

H	19	0.43624	0.00000	0.55972	0.00404	0.56376
* Total *		0.00000	11.99652	39.90685	0.09663	52.00000

Energy: E(RB+HF-LYP) -290.23933191 a.u.

4. Tetramethylammonium Formate

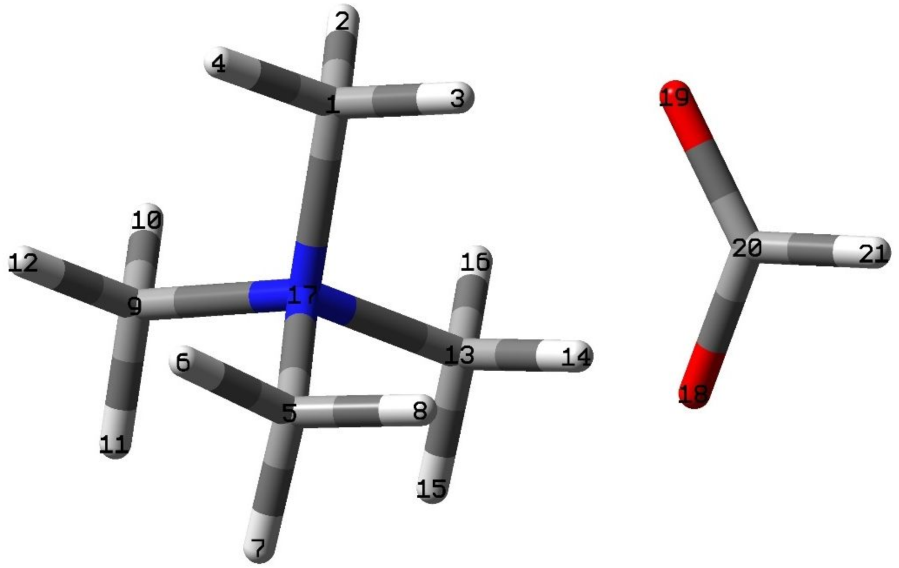

Figure S7. Numbering scheme for atoms in tetramethylammonium formate corresponding to the tables with geometric coordinates and NPA charges.

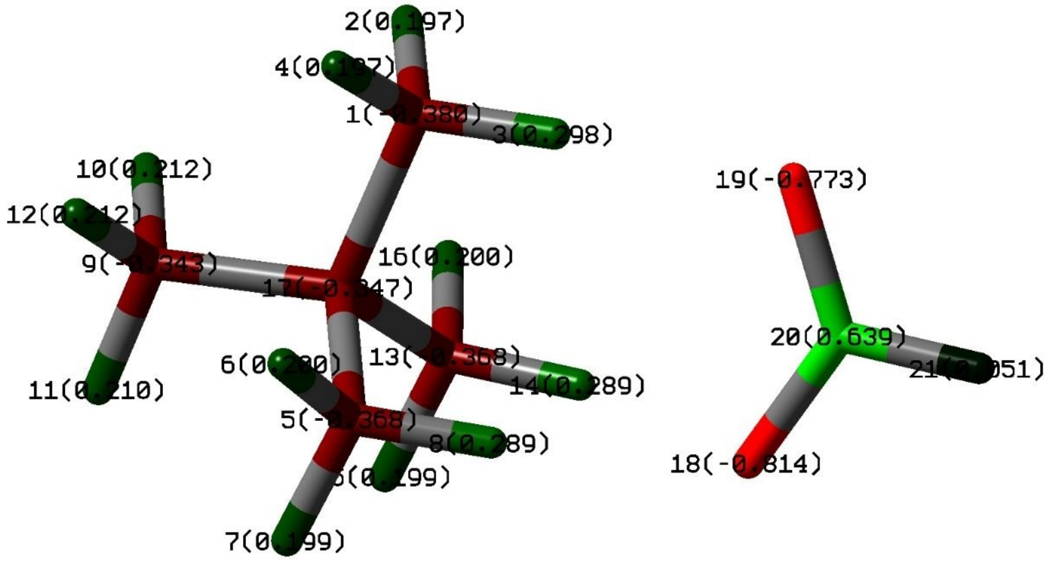

Figure S8. Numbering scheme for atoms in tetramethylammonium formate, NPA charges in parentheses, RED =partial negative charge, GREEN = partial positive charge.

Table S7 Tetramethylammonium Formate - Atomic Coordinates

Standard orientation:

Center Number	Atomic Number	Atomic Type	Coordinates (Angstroms) X	Y	Z
1	6	0	-0.683566	1.407718	-0.002389
2	1	0	-1.037879	1.922836	-0.895625
3	1	0	0.418423	1.327299	-0.002592
4	1	0	-1.037089	1.925603	0.889558
5	6	0	-0.796547	-0.721414	1.226397
6	1	0	-1.066506	-0.137873	2.106030
7	1	0	-1.289811	-1.693255	1.252967
8	1	0	0.287111	-0.848600	1.126999
9	6	0	-2.767351	0.100031	0.000616
10	1	0	-3.094468	0.633547	-0.891226
11	1	0	-3.182809	-0.907259	0.002359
12	1	0	-3.093599	0.636243	0.891158
13	6	0	-0.797756	-0.725113	-1.224615
14	1	0	0.286058	-0.851681	-1.126073
15	1	0	-1.290728	-1.697196	-1.247548
16	1	0	-1.068964	-0.144459	-2.105778
17	7	0	-1.275170	0.017520	0.000004
18	8	0	1.977516	-1.110886	-0.000191
19	8	0	2.317484	1.116549	0.000216
20	6	0	2.700910	-0.075522	-0.000057
21	1	0	3.802316	-0.247346	-0.000160

Table S8 Tetramethylammonium Formate – Natural Population Charges

Summary of Natural Population Analysis:

Atom	No	Natural Charge	Natural Population Core	Valence	Rydberg	Total
C	1	-0.37955	1.99927	4.36453	0.01576	6.37955
H	2	0.19664	0.00000	0.80219	0.00117	0.80336
H	3	0.29809	0.00000	0.69697	0.00494	0.70191
H	4	0.19667	0.00000	0.80215	0.00117	0.80333
C	5	-0.36810	1.99928	4.35378	0.01503	6.36810
H	6	0.20021	0.00000	0.79864	0.00115	0.79979
H	7	0.19904	0.00000	0.79981	0.00116	0.80096
H	8	0.28851	0.00000	0.70822	0.00327	0.71149
C	9	-0.34258	1.99930	4.33008	0.01320	6.34258
H	10	0.21239	0.00000	0.78634	0.00127	0.78761
H	11	0.21046	0.00000	0.78825	0.00130	0.78954
H	12	0.21240	0.00000	0.78633	0.00127	0.78760
C	13	-0.36812	1.99928	4.35381	0.01503	6.36812
H	14	0.28854	0.00000	0.70820	0.00326	0.71146
H	15	0.19905	0.00000	0.79979	0.00116	0.80095

H	16	0.20018	0.00000	0.79867	0.00115	0.79982
N	17	-0.34671	1.99951	5.33788	0.00933	7.34671
O	18	-0.81421	1.99977	6.79980	0.01465	8.81421
O	19	-0.77273	1.99976	6.75809	0.01487	8.77273
C	20	0.63924	1.99972	3.30079	0.06025	5.36076
H	21	0.05059	0.00000	0.94352	0.00589	0.94941
* Total		0.00000	15.99590	49.81783	0.18627	66.00000

Energy: E(RB+HF-LYP) -403.65057310 a.u.

5. Tetramethylammonium nitrite

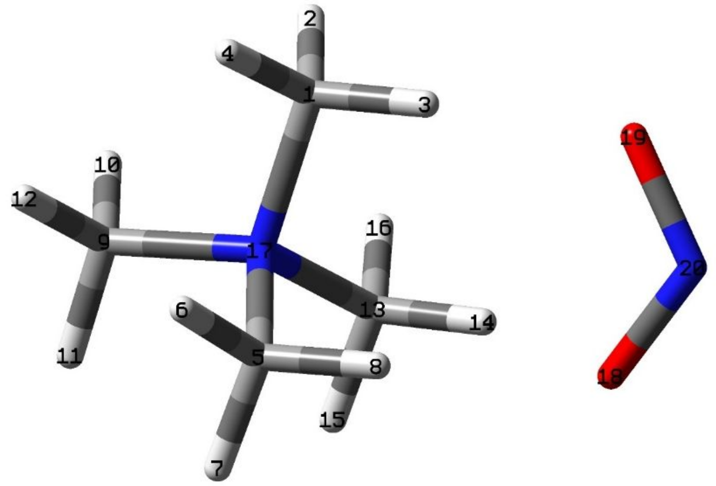

Figure S9 Numbering scheme for atoms in tetramethylammonium nitrite corresponding to the tables with geometric coordinates and NPA charges.

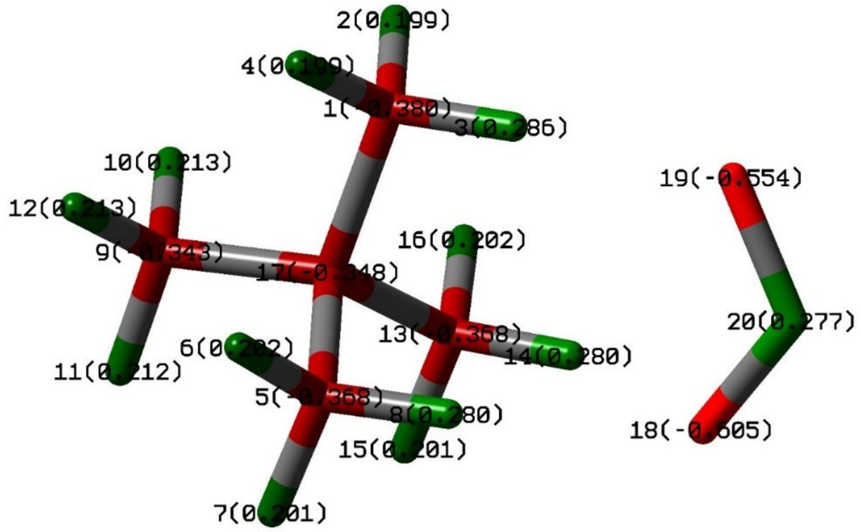

Figure S10 Numbering scheme for atoms in tetramethylammonium nitrite, NPA charges in parentheses, RED =partial negative charge, GREEN = partial positive charge.

Table S9 Tetramethylammonium Nitrite Atomic Coordinates:

Standard orientation:

Center Number	Atomic Number	Atomic Type	Coordinates (Angstroms) X	Y	Z
1	6	0	-0.708552	1.412792	-0.001036
2	1	0	-1.063773	1.926079	-0.894777
3	1	0	0.390389	1.331067	-0.000408
4	1	0	-1.064680	1.927760	0.891383
5	6	0	-0.795990	-0.714224	1.227198
6	1	0	-1.092789	-0.144643	2.107382
7	1	0	-1.257239	-1.701719	1.243361
8	1	0	0.291748	-0.802337	1.140283
9	6	0	-2.778032	0.079164	0.000031
10	1	0	-3.111830	0.608802	-0.891642
11	1	0	-3.178410	-0.934243	0.000583
12	1	0	-3.111774	0.609744	0.891166
13	6	0	-0.796113	-0.716056	-1.226153
14	1	0	0.291697	-0.803601	-1.139351
15	1	0	-1.256985	-1.703757	-1.240505
16	1	0	-1.093440	-0.148057	-2.107180
17	7	0	-1.284512	0.017024	0.000003
18	8	0	2.010734	-1.050574	-0.000182
19	8	0	2.342203	1.052312	-0.000177
20	7	0	2.842471	-0.095460	0.000331

Table S10 Tetramethylammonium Nitrite Natural Population Analysis (NPA) Charges

Summary of Natural Population Analysis:

Atom	No	Natural Charge	Natural Population Core	Valence	Rydberg	Total
C	1	-0.37968	1.99927	4.36426	0.01615	6.37968
H	2	0.19919	0.00000	0.79956	0.00125	0.80081
H	3	0.28568	0.00000	0.70748	0.00684	0.71432
H	4	0.19917	0.00000	0.79958	0.00125	0.80083
C	5	-0.36833	1.99928	4.35344	0.01560	6.36833
H	6	0.20163	0.00000	0.79711	0.00125	0.79837
H	7	0.20135	0.00000	0.79742	0.00124	0.79865
H	8	0.28032	0.00000	0.71488	0.00480	0.71968
C	9	-0.34290	1.99930	4.33051	0.01308	6.34290
H	10	0.21324	0.00000	0.78548	0.00127	0.78676
H	11	0.21177	0.00000	0.78692	0.00130	0.78823
H	12	0.21323	0.00000	0.78549	0.00127	0.78677
C	13	-0.36837	1.99928	4.35348	0.01561	6.36837
H	14	0.28037	0.00000	0.71483	0.00480	0.71963
H	15	0.20134	0.00000	0.79742	0.00124	0.79866

H	16	0.20161	0.00000	0.79714	0.00125	0.79839
N	17	-0.34772	1.99951	5.33759	0.01062	7.34772
O	18	-0.60531	1.99982	6.57936	0.02613	8.60531
O	19	-0.55364	1.99981	6.52862	0.02521	8.55364
N	20	0.27704	1.99979	4.63389	0.08928	6.72296
* Total *		0.00000	15.99608	49.76448	0.23945	66.00000

6. Tetramethylammonium Nitrate (Edge)

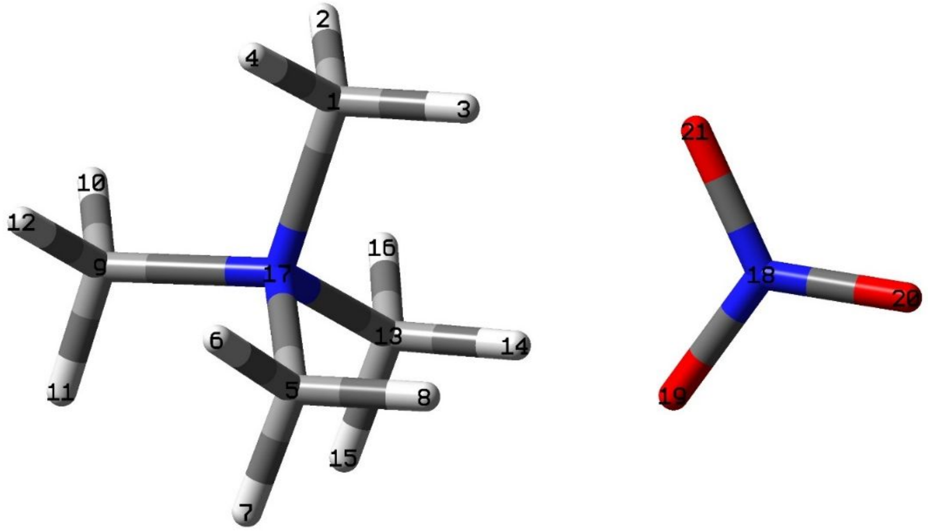

Figure S11 Numbering scheme for atoms in tetramethylammonium nitrite corresponding to the tables with geometric coordinates and NPA charges.

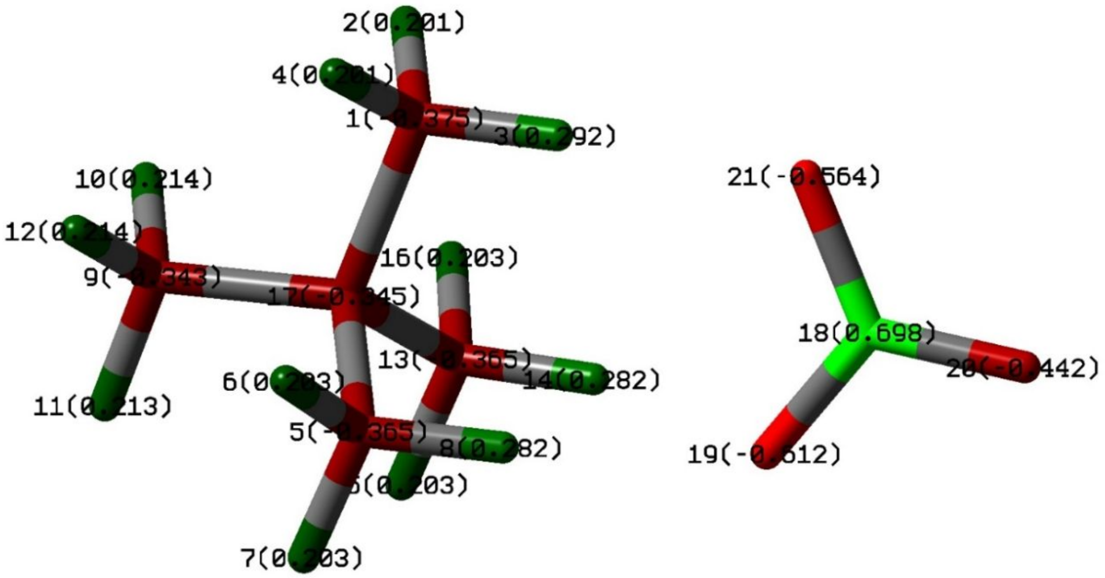

Figure S12 Numbering scheme for atoms in tetramethylammonium nitrite, NPA charges in parentheses, RED =partial negative charge, GREEN = partial positive charge.

Table S11 Tetramethylammonium Nitrate (Edge) Atomic Coordinates

Standard orientation:

Center Number	Atomic Number	Atomic Type	Coordinates (Angstroms) X	Y	Z
1	6	0	-1.153790	1.403045	-0.024454
2	1	0	-1.516207	1.898191	-0.925302
3	1	0	-0.056843	1.342913	-0.022903
4	1	0	-1.515370	1.928570	0.859372
5	6	0	-1.216747	-0.706012	1.237484
6	1	0	-1.526937	-0.131163	2.109439
7	1	0	-1.660689	-1.701019	1.265472
8	1	0	-0.128402	-0.776259	1.160760
9	6	0	-3.208536	0.048872	0.003775
10	1	0	-3.550188	0.562176	-0.894316
11	1	0	-3.599296	-0.967910	0.020501
12	1	0	-3.544578	0.589024	0.888123
13	6	0	-1.224903	-0.745740	-1.217648
14	1	0	-0.135947	-0.812112	-1.147101
15	1	0	-1.667223	-1.741854	-1.209347
16	1	0	-1.542893	-0.200802	-2.105851
17	7	0	-1.713688	0.000852	-0.000249
18	7	0	2.385076	0.002558	-0.000214
19	8	0	1.573485	-0.987863	0.002615
20	8	0	3.599207	-0.185649	-0.002598
21	8	0	1.898397	1.171685	0.001164

Table S12 Tetramethylammonium Nitrate (Edge) Natural Population Analysis (NPA) Charges

Summary of Natural Population Analysis:

Atom	No	Natural Charge	Natural Population Core	Valence	Rydberg	Total
C	1	-0.37465	1.99928	4.35926	0.01611	6.37465
H	2	0.20104	0.00000	0.79781	0.00115	0.79896
H	3	0.29184	0.00000	0.70449	0.00367	0.70816
H	4	0.20118	0.00000	0.79767	0.00115	0.79882
C	5	-0.36511	1.99929	4.35053	0.01530	6.36511
H	6	0.20323	0.00000	0.79563	0.00114	0.79677
H	7	0.20299	0.00000	0.79587	0.00114	0.79701
H	8	0.28242	0.00000	0.71433	0.00325	0.71758

C	9	-0.34334	1.99931	4.33078	0.01325	6.34334
H	10	0.21409	0.00000	0.78469	0.00121	0.78591
H	11	0.21293	0.00000	0.78585	0.00122	0.78707
H	12	0.21412	0.00000	0.78466	0.00121	0.78588
C	13	-0.36514	1.99929	4.35059	0.01526	6.36514
H	14	0.28245	0.00000	0.71435	0.00319	0.71755
H	15	0.20312	0.00000	0.79574	0.00114	0.79688
H	16	0.20309	0.00000	0.79577	0.00115	0.79691
N	17	-0.34452	1.99951	5.33637	0.00864	7.34452
N	18	0.69835	1.99974	4.24182	0.06008	6.30165
O	19	-0.61207	1.99980	6.59740	0.01487	8.61207
O	20	-0.44163	1.99978	6.42641	0.01544	8.44163
O	21	-0.56439	1.99979	6.54966	0.01494	8.56439
* Total *		0.00000	17.99578	55.80970	0.19452	74.00000

7. Tetramethylammonium Nitrate (Face)

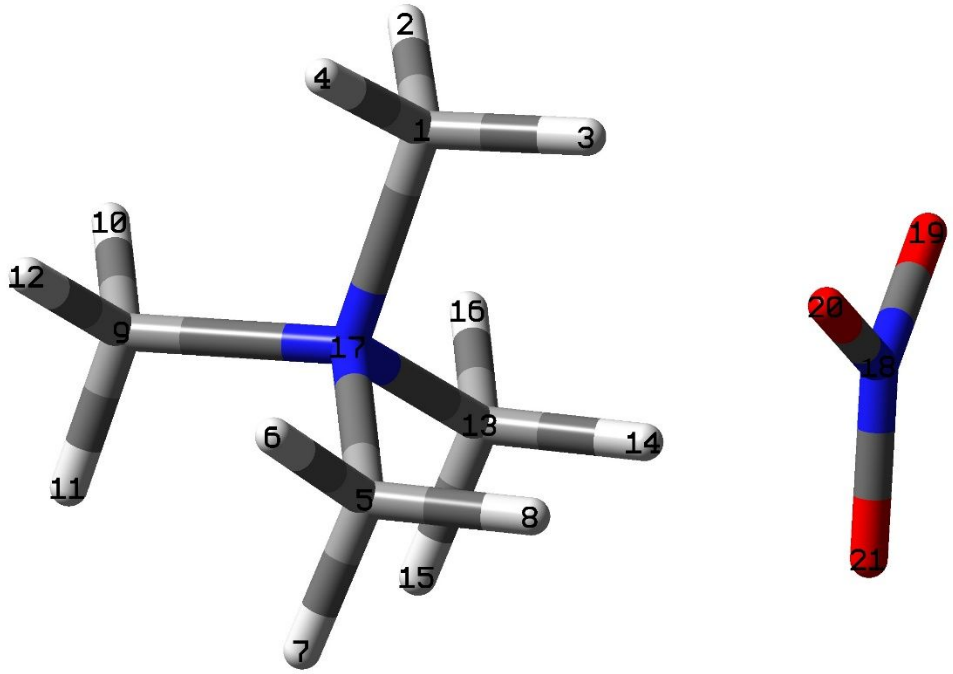

Figure S13 Numbering scheme for atoms in tetramethylammonium nitrate (Face) corresponding to the tables with geometric coordinates and NPA charges.

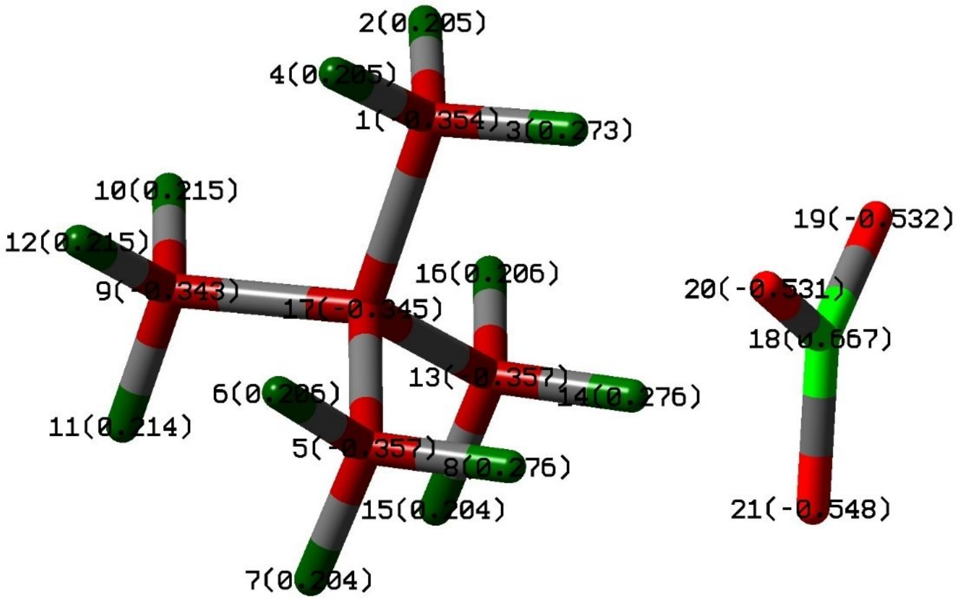

Figure S14 Numbering scheme for atoms in tetramethylammonium nitrate (Face), NPA charges in parentheses, RED =partial negative charge, GREEN = partial positive charge.

Table S13 Tetramethylammonium Nitrate (Face) Atomic Coordinates

TMA Nitrate (Face)

Standard orientation:

Center Number	Atomic Number	Atomic Type	Coordinates (Angstroms) X	Y	Z
1	6	0	-0.925807	1.417976	-0.110653
2	1	0	-1.297352	1.847773	-1.039993
3	1	0	0.162067	1.403091	-0.112015
4	1	0	-1.293816	1.986107	0.742657
5	6	0	-0.985296	-0.624656	1.288293
6	1	0	-1.309156	0.005721	2.115323
7	1	0	-1.438114	-1.612571	1.365769
8	1	0	0.100657	-0.709598	1.272856
9	6	0	-2.956930	0.056109	-0.002445
10	1	0	-3.296629	0.513672	-0.930606
11	1	0	-3.345614	-0.958308	0.073918
12	1	0	-3.294532	0.647724	0.847420
13	6	0	-0.988212	-0.817511	-1.176177
14	1	0	0.098399	-0.893893	-1.154335
15	1	0	-1.433572	-1.808291	-1.091544
16	1	0	-1.322672	-0.330188	-2.090984

17	7	0	-1.459195	0.007980	-0.000408
18	7	0	2.058184	-0.009719	0.000330
19	8	0	2.067179	0.558229	-1.121837

Table S14 Tetramethylammonium Nitrate (Face) Natural Population Analysis (NPA) Charges

Summary of Natural Population Analysis:

Atom	No	Natural Charge	Natural Population Core	Valence	Rydberg	Total
C	1	-0.35404	1.99928	4.33847	0.01629	6.35404
H	2	0.20540	0.00000	0.79346	0.00114	0.79460
H	3	0.27321	0.00000	0.72238	0.00441	0.72679
H	4	0.20536	0.00000	0.79350	0.00114	0.79464
C	5	-0.35695	1.99929	4.34148	0.01619	6.35695
H	6	0.20639	0.00000	0.79248	0.00112	0.79361
H	7	0.20398	0.00000	0.79487	0.00114	0.79602
H	8	0.27587	0.00000	0.71963	0.00450	0.72413
C	9	-0.34307	1.99930	4.33050	0.01327	6.34307
H	10	0.21490	0.00000	0.78388	0.00122	0.78510
H	11	0.21447	0.00000	0.78433	0.00120	0.78553
H	12	0.21492	0.00000	0.78386	0.00122	0.78508
C	13	-0.35731	1.99929	4.34184	0.01618	6.35731
H	14	0.27614	0.00000	0.71937	0.00449	0.72386
H	15	0.20406	0.00000	0.79479	0.00114	0.79594
H	16	0.20616	0.00000	0.79272	0.00112	0.79384
N	17	-0.34511	1.99951	5.33766	0.00794	7.34511
N	18	0.66731	1.99973	4.27069	0.06227	6.33269
O	19	-0.53192	1.99980	6.51800	0.01411	8.53192
O	20	-0.53139	1.99980	6.51749	0.01411	8.53139
O	21	-0.54839	1.99980	6.53448	0.01411	8.54839
* Total *		0.00000	17.99580	55.80588	0.19832	74.00000

8. Tetramethylammonium carbonate Edge

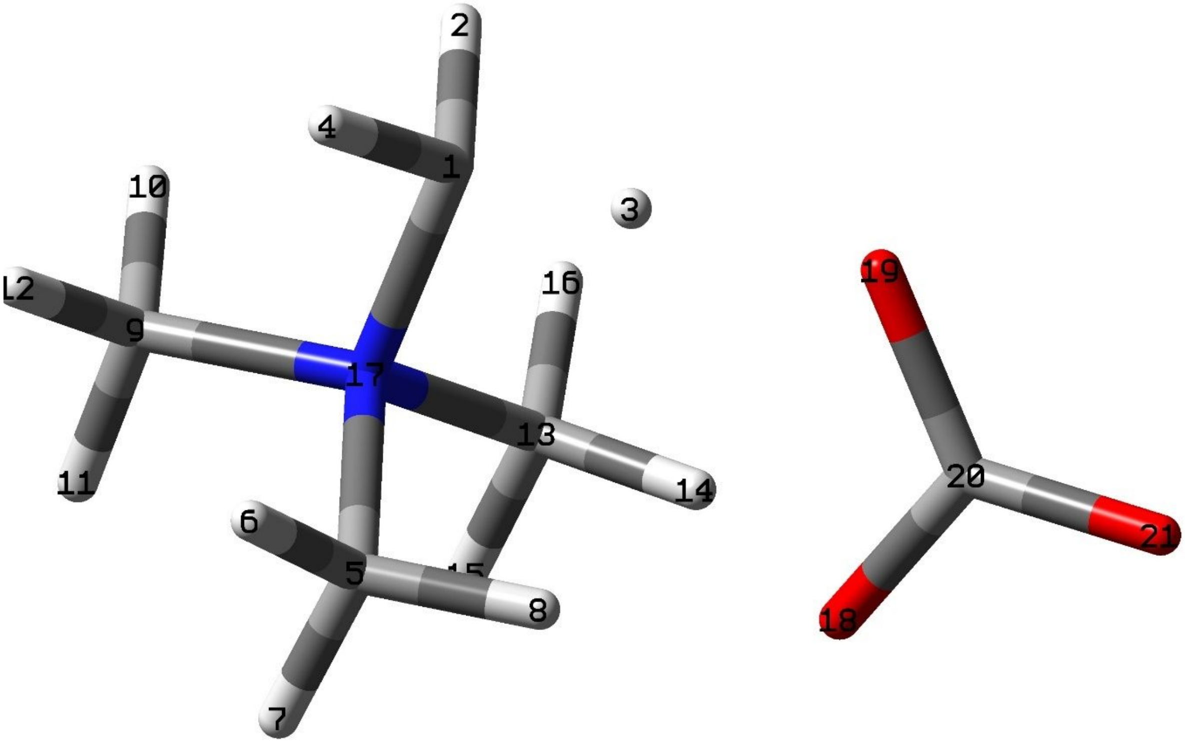

Figure S15 Numbering scheme for atoms in tetramethylammonium carbonate-edge corresponding to the tables with geometric coordinates and NPA charges.

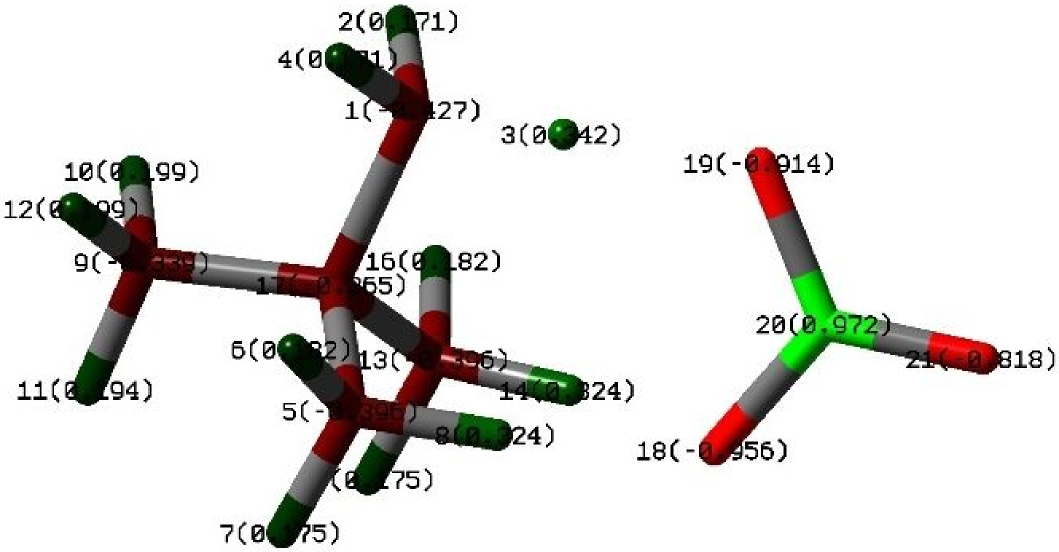

Figure S16 Numbering scheme for atoms in tetramethylammonium nitrite, NPA charges in parentheses, RED =partial negative charge, GREEN = partial positive charge.

Table S15 Tetramethylammonium Carbonate (Edge) Atomic Coordinates

Standard orientation:

Center Number	Atomic Number	Atomic Type	Coordinates (Angstroms) X	Y	Z
1	6	0	-0.993591	1.395869	-0.002560
2	1	0	-1.361459	1.905105	-0.897617
3	1	0	0.177533	1.298764	-0.001988
4	1	0	-1.361636	1.908572	0.890448
5	6	0	-1.088369	-0.727155	1.223944
6	1	0	-1.336364	-0.127717	2.101323
7	1	0	-1.592650	-1.695580	1.269443
8	1	0	0.012719	-0.853107	1.058253
9	6	0	-3.078026	0.063004	0.000700
10	1	0	-3.413198	0.593153	-0.891714
11	1	0	-3.485045	-0.950225	0.002449
12	1	0	-3.412356	0.595848	0.891821
13	6	0	-1.089577	-0.731317	-1.221769
14	1	0	0.011804	-0.856161	-1.057035
15	1	0	-1.593356	-1.700187	-1.263041
16	1	0	-1.339139	-0.135261	-2.101018
17	7	0	-1.590812	0.004347	0.000080
18	8	0	1.443225	-1.033473	-0.000018
19	8	0	1.733307	1.197986	-0.000057
20	6	0	2.283350	0.001273	-0.000075
21	8	0	3.526732	-0.167474	-0.000340

Table S16: Natural Population Analysis Charges:

Summary of Natural Population Analysis:

Atom	No	Natural Charge	Natural Population Core	Valence	Rydberg	Total
C	1	-0.42681	1.99928	4.40470	0.02283	6.42681
H	2	0.17057	0.00000	0.82785	0.00159	0.82943
H	3	0.34227	0.00000	0.65292	0.00481	0.65773
H	4	0.17058	0.00000	0.82784	0.00159	0.82942
C	5	-0.39593	1.99928	4.37787	0.01878	6.39593
H	6	0.18233	0.00000	0.81615	0.00152	0.81767
H	7	0.17533	0.00000	0.82322	0.00145	0.82467
H	8	0.32425	0.00000	0.67140	0.00435	0.67575
C	9	-0.33936	1.99930	4.32619	0.01386	6.33936

H	10	0.19902	0.00000	0.79902	0.00196	0.80098
H	11	0.19368	0.00000	0.80416	0.00216	0.80632
H	12	0.19902	0.00000	0.79901	0.00196	0.80098
C	13	-0.39595	1.99928	4.37790	0.01878	6.39595
H	14	0.32428	0.00000	0.67138	0.00434	0.67572
H	15	0.17536	0.00000	0.82319	0.00145	0.82464
H	16	0.18227	0.00000	0.81621	0.00152	0.81773
N	17	-0.36462	1.99950	5.34870	0.01642	7.36462
O	18	-0.95632	1.99977	6.94278	0.01376	8.95632
O	19	-0.91361	1.99976	6.89993	0.01391	8.91361
C	20	0.97157	1.99972	2.96163	0.06708	5.02843
O	21	-0.81793	1.99975	6.80562	0.01257	8.81793
* Total *		-1.00000	17.99565	55.77766	0.22668	74.00000

9. Tetramethylammonium Carbonate Face

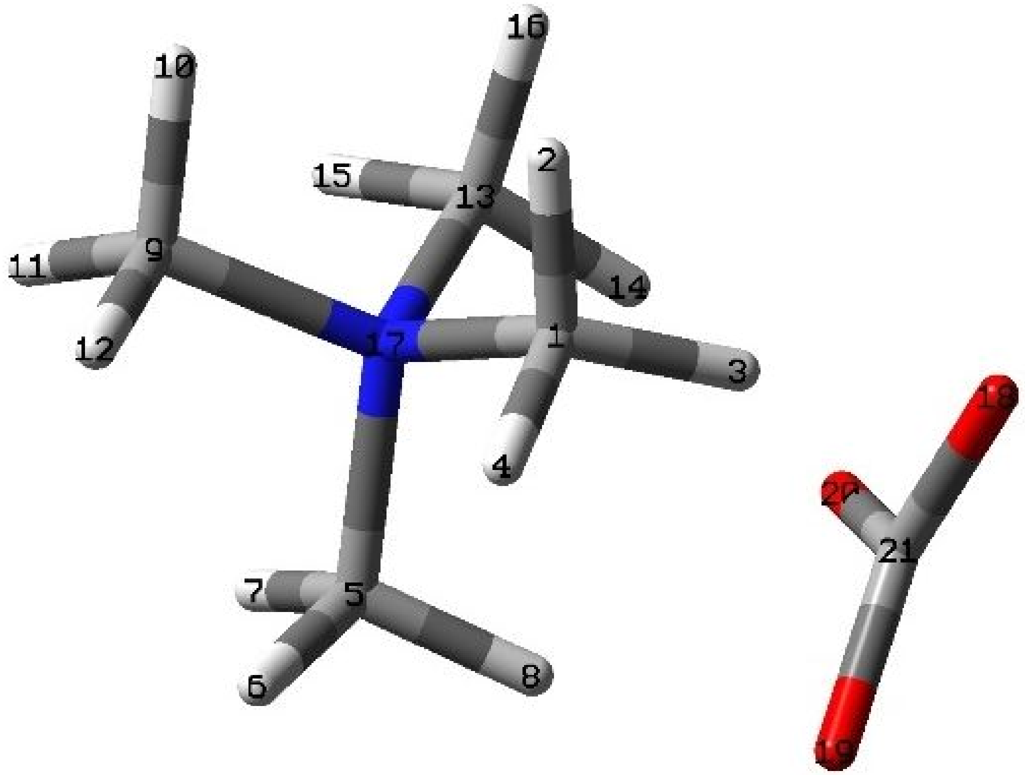

Figure S17 Numbering scheme for atoms in tetramethylammonium carbonate-face corresponding to the tables with geometric coordinates and NPA charges.

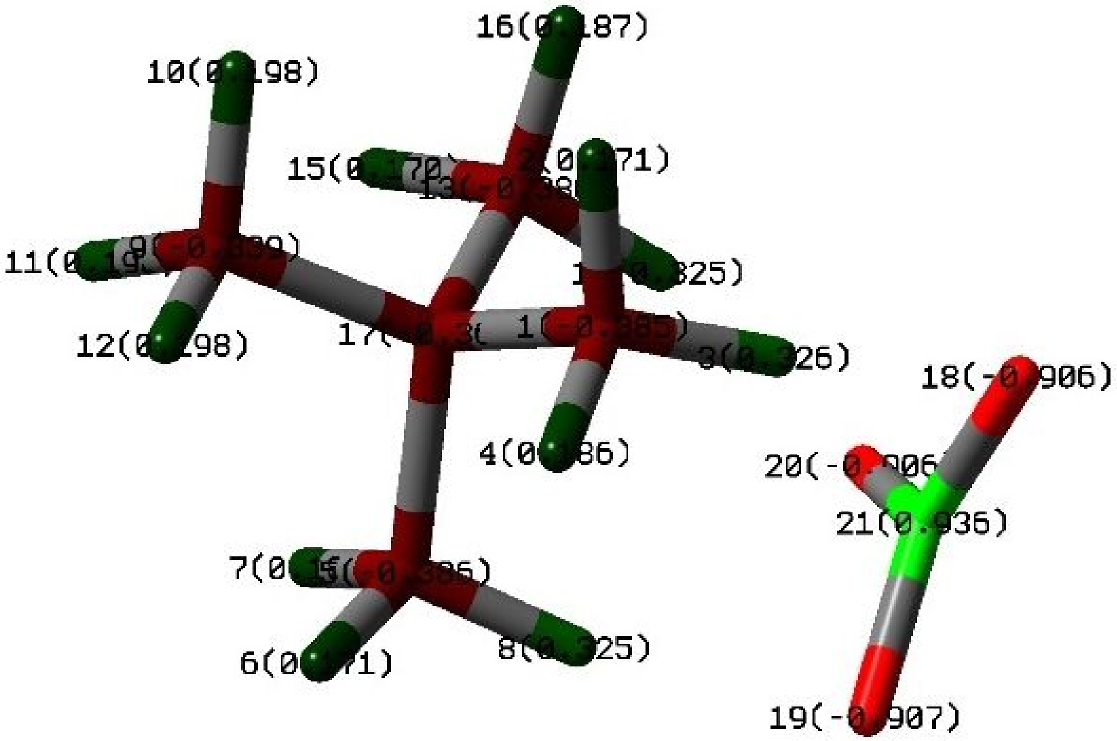

Figure S18 Numbering scheme for atoms in tetramethylammonium carbonate facing, NPA charges in parentheses, RED =partial negative charge, GREEN = partial positive charge.

Table S17: Atomic Coordinates for tetramethylammonium carbonate-face:

Standard orientation:

Center Number	Atomic Number	Atomic Type	Coordinates (Angstroms) X	Y	Z
1	6	0	0.844073	-1.302225	-0.591626
2	1	0	1.383131	-1.459043	-1.529454
3	1	0	-0.257671	-1.226199	-0.780394
4	1	0	1.077329	-2.097867	0.117291
5	6	0	0.851015	0.137659	1.425828
6	1	0	1.393984	-0.595075	2.028377
7	1	0	1.082199	1.149844	1.761180
8	1	0	-0.250488	-0.067081	1.456812
9	6	0	2.836950	0.001714	-0.007363
10	1	0	3.190851	-0.031373	-1.038499
11	1	0	3.197765	0.910335	0.476248
12	1	0	3.199386	-0.872562	0.534763
13	6	0	0.841516	1.166866	-0.828238
14	1	0	-0.256283	1.301822	-0.650322

15	1	0	1.394530	2.053783	-0.507853
16	1	0	1.052855	0.946170	-1.875324
17	7	0	1.346032	0.000772	-0.000637
18	8	0	-1.918761	-0.656077	-1.121475
19	8	0	-1.909607	-0.646248	1.128479
20	8	0	-1.926988	1.298065	-0.004979
21	6	0	-1.971382	-0.001360	0.000639

Table S18. Tetramethylammonium carbonate-face NPA Charges

Summary of Natural Population Analysis:

Atom	No	Natural Charge	Natural Population Core	Valence	Rydberg	Total
C	1	-0.38546	1.99927	4.36681	0.01939	6.38546
H	2	0.17088	0.00000	0.82758	0.00153	0.82912
H	3	0.32550	0.00000	0.66923	0.00527	0.67450
H	4	0.18561	0.00000	0.81298	0.00141	0.81439
C	5	-0.38604	1.99927	4.36743	0.01934	6.38604
H	6	0.17117	0.00000	0.82730	0.00153	0.82883
H	7	0.18544	0.00000	0.81315	0.00141	0.81456
H	8	0.32543	0.00000	0.66933	0.00524	0.67457
C	9	-0.33877	1.99930	4.32564	0.01382	6.33877
H	10	0.19847	0.00000	0.79971	0.00182	0.80153
H	11	0.19832	0.00000	0.79986	0.00182	0.80168
H	12	0.19843	0.00000	0.79975	0.00182	0.80157
C	13	-0.38562	1.99927	4.36697	0.01938	6.38562
H	14	0.32476	0.00000	0.67003	0.00521	0.67524
H	15	0.17045	0.00000	0.82801	0.00154	0.82955
H	16	0.18700	0.00000	0.81160	0.00141	0.81300
N	17	-0.36358	1.99951	5.34855	0.01552	7.36358
O	18	-0.90566	1.99977	6.89238	0.01351	8.90566
O	19	-0.90657	1.99977	6.89332	0.01348	8.90657
O	20	-0.90563	1.99977	6.89238	0.01348	8.90563
C	21	0.93585	1.99970	2.98957	0.07487	5.06415
* Total *		-1.00000	17.99563	55.77159	0.23279	74.00000

E(RB+HF-LYP) -478.29883419 a.u.

10. TMA Nitromethane

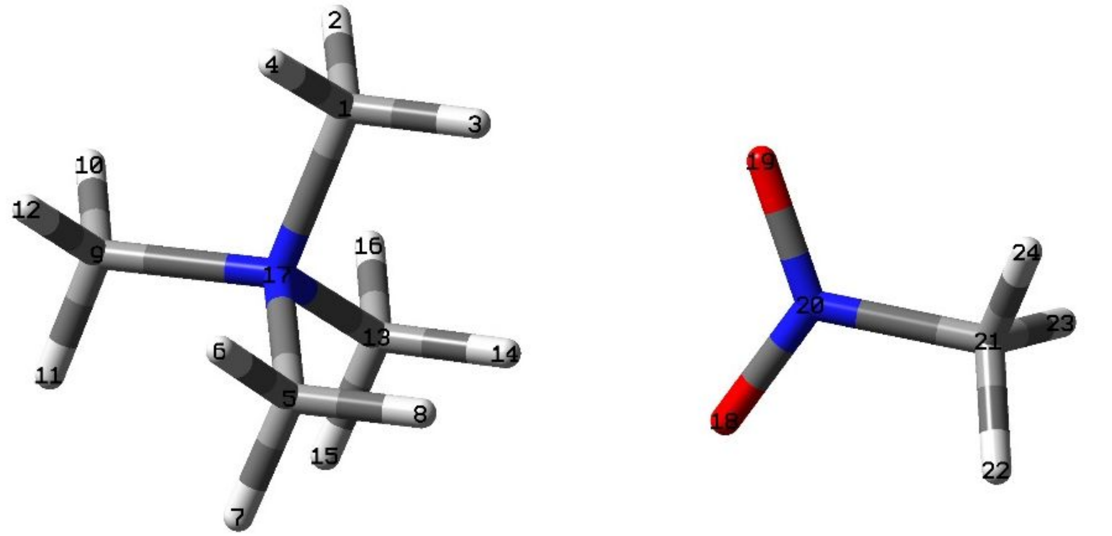

Figure S19 Numbering scheme for atoms in the tetramethylammonium nitromethane polar group-cation pair corresponding to the tables with geometric coordinates and NPA charges.

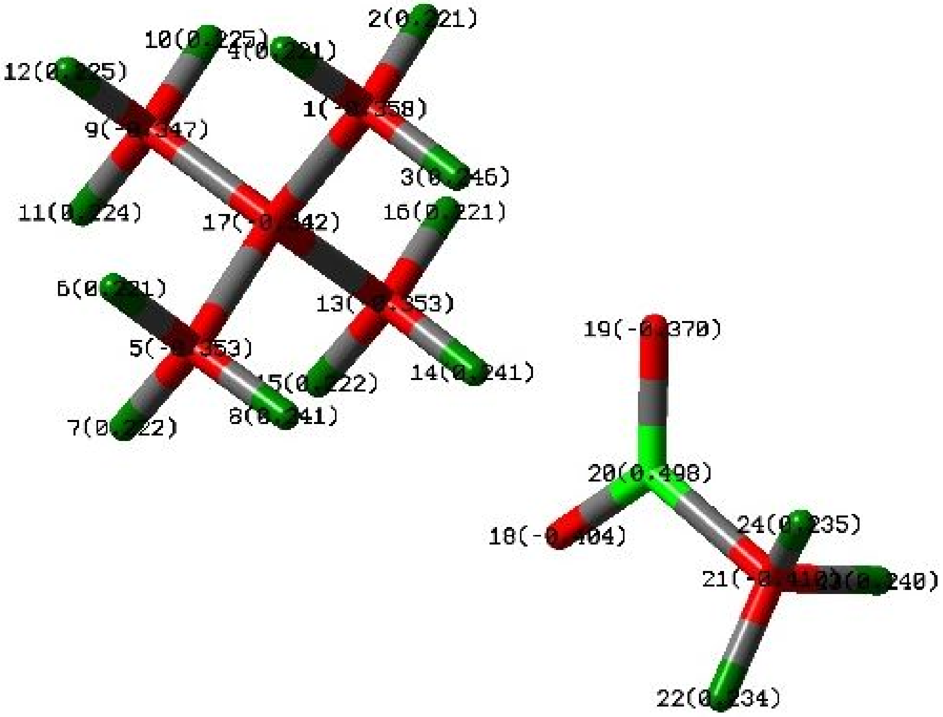

Figure S20 Numbering scheme for atoms in the tetramethylammonium nitromethane polar group-cation pair, NPA charges in parentheses, RED = partial negative charge, GREEN = partial positive charge.

TMA nitromethane

Table S19: Atomic Coordinates for tetramethylammonium nitromethane polar group-cation pair

Standard orientation:

Center Number	Atomic Number	Atomic Type	Coordinates (Angstroms) X	Y	Z
1	6	0	1.408900	1.392839	0.000406
2	1	0	1.758279	1.911440	0.892012
3	1	0	0.321459	1.331735	-0.000456
4	1	0	1.759822	1.911291	-0.890676
5	6	0	1.504787	-0.738987	-1.227573
6	1	0	1.830916	-0.191747	-2.110629
7	1	0	1.939065	-1.737658	-1.226830
8	1	0	0.418944	-0.802960	-1.193429
9	6	0	3.481646	0.063586	0.001973
10	1	0	3.810887	0.594940	0.893529
11	1	0	3.882391	-0.948849	0.002895
12	1	0	3.812031	0.593777	-0.889853
13	6	0	1.503134	-0.738342	1.229343
14	1	0	0.417133	-0.799987	1.195182
15	1	0	1.935372	-1.737900	1.228458
16	1	0	1.830291	-0.191954	2.112551
17	7	0	1.978777	-0.004429	0.001026
18	8	0	-1.774234	-0.964526	-0.006170
19	8	0	-2.071364	1.178611	-0.003129
20	7	0	-2.491634	0.031667	-0.012146
21	6	0	-3.976318	-0.175119	0.005409
22	1	0	-4.188645	-1.116459	-0.492999
23	1	0	-4.268826	-0.223140	1.055089
24	1	0	-4.437221	0.680263	-0.479957

Table S20. Tetramethylammonium nitromethane polar group-cation pair NPA Charges

Summary of Natural Population Analysis:

Atom	No	Natural Charge	Natural Population Core	Valence	Rydberg	Total
C	1	-0.35761	1.99929	4.34362	0.01470	6.35761
H	2	0.22060	0.00000	0.77835	0.00105	0.77940
H	3	0.24632	0.00000	0.75029	0.00339	0.75368
H	4	0.22061	0.00000	0.77834	0.00105	0.77939
C	5	-0.35268	1.99930	4.33902	0.01437	6.35268
H	6	0.22098	0.00000	0.77796	0.00106	0.77902
H	7	0.22155	0.00000	0.77741	0.00104	0.77845
H	8	0.24117	0.00000	0.75661	0.00223	0.75883
C	9	-0.34696	1.99930	4.33368	0.01398	6.34696
H	10	0.22473	0.00000	0.77420	0.00107	0.77527
H	11	0.22417	0.00000	0.77475	0.00108	0.77583

H	12	0.22476	0.00000	0.77418	0.00107	0.77524
C	13	-0.35268	1.99930	4.33903	0.01435	6.35268
H	14	0.24114	0.00000	0.75661	0.00225	0.75886
H	15	0.22156	0.00000	0.77739	0.00104	0.77844
H	16	0.22092	0.00000	0.77802	0.00106	0.77908
N	17	-0.34227	1.99951	5.33471	0.00805	7.34227
O	18	-0.40379	1.99979	6.38810	0.01590	8.40379
O	19	-0.37032	1.99978	6.35467	0.01586	8.37032
N	20	0.49831	1.99962	4.44731	0.05476	6.50169
C	21	-0.40992	1.99926	4.39930	0.01135	6.40992
H	22	0.23401	0.00000	0.76463	0.00136	0.76599
H	23	0.23994	0.00000	0.75897	0.00109	0.76006
H	24	0.23545	0.00000	0.76318	0.00137	0.76455
Total		1.00000	17.99515	55.82035	0.18450	74.00000

Table S21: Calculated Energies for the structures in Hartrees (Atomic Units):

	Compound	Energy (a.u.)
1	Tetramethylammonium (TMA) cation	-214.22319864
2	TMA Chloride	-674.60482242
3	TMA Hydroxide	-290.23933191
4	TMA Formate	-403.65057310
5	TMA Nitrite	-419.596434737
6	TMA Nitrate Edge	-494.819632481
7	TMA Nitrate Face	-494.815658351
8	TMA Carbonate Edge	-478.302043505
9	TMA Carbonate Face	-478.29883419
10	TMA Nitromethane	-459.333448460
